# Metagenomic analysis of antimicrobial resistance genes in domestic canines

**DOI:** 10.1016/j.onehlt.2026.101380

**Published:** 2026-03-04

**Authors:** Hillary A. Craddock, Yair Motro, Katherine M. Winner, Yuval Lotem-Michaeli, Eran Segal, Anastasia Godneva, Dan Grinstein, Jacob Moran-Gilad

**Affiliations:** a Department of Health Policy and Management, School of Public Health, Faculty of Health Sciences, Ben Gurion University of the Negev, 1 Ben Gurion Blvd, Building 39, Beer Sheva 8410501, Israel; b Department of Microbiology and Immunology, University of Michigan Medical School, 1500 E. Medical Center Dr., Ann Arbor, MI 48109, USA; c Department of Computer Science and Applied Mathematics, Weizmann Institute of Science, 234 Herzl Street, POB 26, Rehovot 7610001, Israel; d Mohamed bin Zayed University of Artificial Intelligence, Building 1B, Masdar City, Abu Dhabi, United Arab Emirates; e Department of Molecular Cell Biology, Weizmann Institute of Science, 234 Herzl Street, Rehovot 7610001, Israel

**Keywords:** Antibiotic resistance, One health, Environmental health, Genomics, Working dogs, Zoonoses

## Abstract

A One Health approach is critical to addressing the spread of antimicrobial resistance (AMR). A key source of AMR in humans is companion animals, particularly canines. Recent investigation has shown that the canine fecal microbiome is rich in antimicrobial resistant genes (ARGs), yet few studies have studied the resistome of working canines. Our objective was to investigate the resistome of canines to elucidate associations between various exposures and demographic factors and ARG carriage. We performed resistome and microbiome analyses on previously-generated metagenomic sequence data from 126 Israeli working canines and 147 global canines. We found that the canine microbiome and resistome varied significantly with country of origin, and the resistome varied significantly with gastrointestinal disease state, canine job type, and microbiome composition. Tetracycline resistant genes were the most dominant across all canines. Extended-spectrum beta lactamase (ESBL) genes were observed in up to 33% of canines. Genes of concern, including potential carbapenemases (*bla*OXA-181 and *bla*OXA-347) and colistin resistance genes (*mcr-10*) were infrequently observed. The *Inc* family of plasmids, typically associated with ESBL genes, were frequently detected. Altogether our research suggests that canines, including working dogs, are a potential source of ARGs and plasmids which carry ARGs. Importantly, the abundance and identity of these ARGs is associated with various potentially modifiable factors such as microbiome composition. As canines are an important human exposure within the One Health paradigm, future work is necessary to understand the risk and transmission dynamics of ARGs between humans and their companion canines.

## 1 Introduction

Elucidating the sources of clinically-relevant antimicrobial resistance (AMR) is both challenging and critical to managing and controlling the emergence and circulation of AMR among human populations. One key potential source of AMR are the animals that interact with human spaces, including livestock, domesticated animals like canines and felines, and wildlife. This falls within the One Health paradigm, which seeks to investigate human, animal, and environmental systems holistically. It is known that canines, both domestic and feral, can carry a wide variety of antibiotic resistant bacteria (ARBs) and antimicrobial resistance genes (ARGs), including but not limited to genes encoding resistance to aminoglycosides, beta lactams, and tetracycline [1], [2]. While numerous studies have investigated ARBs in domestic canines, there are relatively few studies investigating the collective presence of ARGs in canine samples, otherwise known as the canine resistome. Additionally, there is a notable research gap with respect to AMR in working dogs.

Many ARGs commonly identified in canine populations are associated with Mobile Genetic Elements (MGEs), including a study of Israeli domestic dogs noting extended-spectrum beta-lactamase (ESBL) genes and plasmids [3], [4]. Some studies have investigated shared ARGs between canines and humans. For example, Røken et al. noted that the genes *tet(M)* (tetracycline resistance), *ermB* (erythromycin resistance), and *bla*TEM (beta-lactam resistance) were most closely related in terms of prevalence between dogs and humans. This study also noted that *mecA* (encoding methicillin resistance in staphylococci) and various aminoglycoside resistance genes were generally more prevalent among canines than humans, however when household relationships were investigated, *ermF, sul1, tetB, mecA* and two aminoglycoside resistance genes (*ant(3′)* and *aph(3′)*) were likely to be shared in the household [2].

Generally, exposure and demographic factors can influence AMR carriage. For example, some studies have noted that exposures such as food (e.g. raw meat, dry kibble), walking in forested areas, coprophagia, and recent treatment with antibiotics and/or admittance to a veterinary hospital as associated with ESBL carriage in dogs [1], [4], [5], [6], [7]. It has been noted that while the presence of ARBs increases during treatment with antibiotics, and it can return to baseline after several months, certain ARGs such as *bla*CTX-M genes can persist [8]. It should be noted, however, that many studies focus on laboratory or companion dogs. Few studies have focused on stray dogs; for example, in a study comparing companion dogs and stray dogs in Argentina, stray dogs had a higher burden of ESBL-producing *E. coli* than companion dogs [9]. Overall, data on working dogs are scarce.

The goal of this study was to investigate the resistome of working dogs using a metagenomic approach and compare these findings to a global dataset of domestic canine metagenomes to understand the presence of clinically-relevant ARGs as well as to compare resistomes among diverse dog populations. Such an approach is expected to provide a greater understanding of the potential for spillover of AMR between canines and humans.

## 2 Methods

### 2.1 Israeli canine data collection

Data analysis was conducted on canine metagenomic samples from previously published work, including an Israeli cohort. In brief, Israeli samples were collected from freshly voided feces, and as such this study was deemed exempt by the Ben Gurion University of the Negev animal research committee. Samples were collected with Eswabs (Copan Diagnostics, Brescia, Italy) in such a manner as to reduce environmental contamination, as described in Craddock et al. [10]. Dogs receiving antibiotics in the preceding four weeks were excluded. Samples were snap frozen and kept at —80C until processing. DNA was extracted using the PowerMicrobial/PowerSoil DNA kits (Qiagen, Hilden, Germany). Metadata were collected including basic demographic information (e.g. sex, sterilization status, operational job, breed) and factors which are known to influence microbiota composition in canines (antibiotic and proton pump inhibitor (PPI) administration history, diet, history of gastrointestinal disorders). The datasets from this study are available in a BioProject repository, under project number PRJEB45252.

### 2.2 Global data collection

A total of 147 publicly available non-duplicate metagenome sequences were retrieved; these were publicly available as raw sequence data from the National Center for Biotechnology Information (NCBI) Sequence Read Archive database. Samples were selected based on metagenomic data availability of healthy canines, while studies including diseased canines or studies limited to 16S *rRNA* amplicon sequencing methodologies were excluded. Geographic, breed, and related metadata available from NCBI for downloaded genomes were obtained for later clustering analysis. Further details on collection and ethical approvals for the other studies are available in the original papers [11], [12].

### 2.3 Data analysis

In total, 273 samples underwent QC using fastp, and taxonomic profiling was carried out on the QC reads using kraken2 with the refseq100GB database. ARGs profiling was performed using ARGs_OAP and reads were then assembled using Megahit. The resistomes and plasmidomes were predicted using Abricate, AMRFinderPlus, the CARD database, the MEGARes database, and the plasmidfinder database. The metagenome assembled genomes (MAGs) were isolated using SemiBin, minimap2, and samtools. The taxonomic assignments of the MAGs were determined using GTDBTk. The MLST, resistome, and plasmidome of the good quality and dereplicated MAGs were determined using MLST, Abricate, AMRFinderPlus, the CARD database, the MEGARes database, and the plasmidfinder database. Citations and further details are available in Supplemental Methods.

Microbial community analysis was performed in R (version 4.3.0) [13] and utilized the tidyverse [14], ggplot2 [15], vegan [16], and microeco [17] packages. The *microeco* package was used in R to normalize samples by total sum scaling and perform relative abundance calculations, and a random forest classification model with Mean Decrease in Gini was used as an index of feature importance to determine differential abundance of microbial communities and ARGs across samples using the *microeco* package in R. All *P* values are false discovery rate-adjusted.

## 3 Results

### 3.1 Global cohort demographics and microbiome composition

Overall, 273 canine metagenomes were included in the study, with the largest proportion consisting of samples originating from working dogs from Israel (*n* = 126) and laboratory dogs from the United States (US) (*n* = 66). Of collected demographic information, only breed differed significantly by location (*P* < 0.0001). Further information is available in Supplemental Results (inc. Supplemental Table 1).

### 3.2 Gut microbiome analysis

We performed microbiome analysis on samples from 273 dogs. Bacterial taxa varied largely in their proportions between individuals; 49 phyla were found across all samples, but only 6 phyla had abundance >0.1% and accounted for more than 99% of relative abundance (Supplemental Fig. 1 and Supplemental Table 2). The most abundant bacterial phyla were the *Bacteroidota* (50.2% ± 31.0%) and *Bacillota* (32.7% ± 23.2%).

**Fig. 1:**
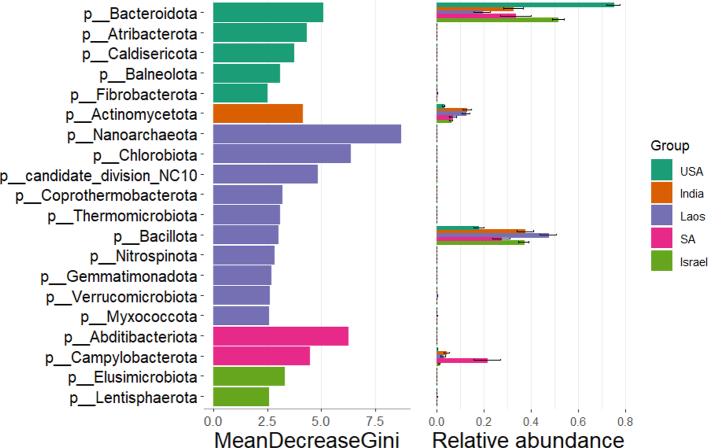
Random forest analysis identifies *Bacteroidota*, *Actinomycetoma*, *Bacillota*, and *Campylobacterota* as differentially abundant among domestic canine samples across countries of origin. * SA = South Africa. USA = United States of America.

Country of origin was found to be significantly associated with alpha-diversity. Dogs from Laos (mostly shelter and street dogs) had a significantly higher Shannon index than dogs from any other country (*P* < 0.0001, Wilcoxon Rank Sum). Conversely, dogs from the US, which represent working and laboratory dogs, had a significantly lower Shannon index than dogs from any other country (P < 0.0001, Wilcoxon Rank Sum). This suggests higher within-sample diversity among dogs from Laos and lower within-sample diversity among dogs from the US. Beta diversity refers to the similarity or dissimilarity between communities, indicating the overlap in microbial composition between sample pairs. Beta diversity was compared between groups using the Bray-Curtis dissimilarity index. A significant difference in beta diversity was observed by canine country of origin (for all comparisons *P* ≤ 0.02, Wilcoxon Rank Sum). Differential abundance was determined by Random Forest to identify community differences across groups. *Campylobacterota* was more abundant in the global canine population compared to the Israeli canine population; this bacterial phylum could be the driver of the difference in beta diversity that was demonstrated between these groups. (Fig. 1).

Further results of microbiome comparisons are presented in the Supplementary Materials (inc. Supplemental Tables 3 & 4, Supplemental Figs. 2, 3, 4, & 5). More in-depth analyses of these canine microbiomes in individual studies are available in the original papers [10], [11], [12].

### 3.3 Resistome results - global ARG trends

**Fig. 2:**
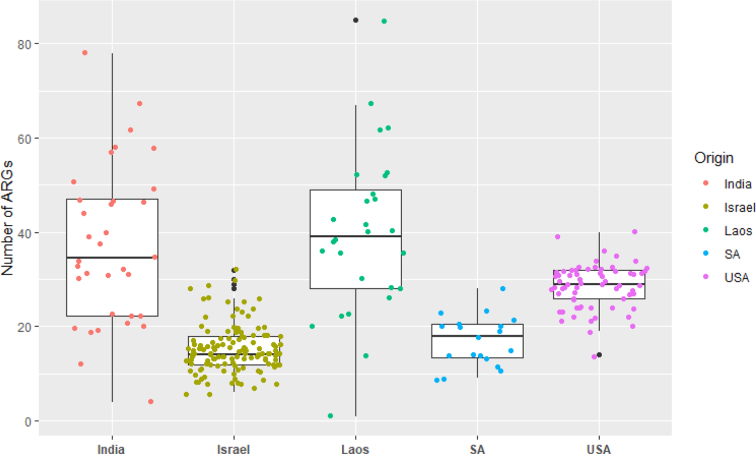
Number of antimicrobial resistance genes (ARGs) in canine samples by country of origin. * SA = South Africa, USA = United States of America.

From the 273 metagenomes, we identified 217 ARGs among one or more dogs, and their prevalence is presented in Supplemental Table 5. There was a median of 24.5 (IQR: 24.75) ARGs identified in each dog (Mean: 32.2 genes, Range: 1–85 genes). Of note, in one dog we detected just a single ARG, *Inu(C)*. The total number of ARGs varied significantly by country of origin (Fig. 2). ARGs identified in the dogs were related to 25 antimicrobial classes, but only 9 antimicrobial classes had ARG abundance >1% and these 9 classes accounted for 97.59% of all ARGs (Supplemental Table 6, Supplemental Figs. 6). In non-normalized ARG counts, the Israeli and US dog ARG counts clustered closely together compared to other origins; this may reflect that these dogs were the most tightly controlled of these groups, as they are working dogs and laboratory dogs, respectively (Fig. 2). Overall, tetracycline genes were the most dominant (31.7% relative abundance) across all dogs in the cohort. Macrolide (16.0% relative abundance), lincosamide (14.5% relative abundance), aminoglycoside (13.9% relative abundance), and beta-lactam (12.6% relative abundance) ARGs were among the most abundant in all dogs. This pattern was generally observed among the different countries (Supplemental Fig. 6). Relative abundance by class did not vary significantly by geographic origin (Supplemental Fig. 7). Alpha diversity (Supplemental Figs. 8 & 9) and Beta diversity (Supplemental Figs. 10 & 11) did differ by geographic origin.

### 3.4 Israeli Dog Resistome - ARG Trends.

**Fig. 3:**
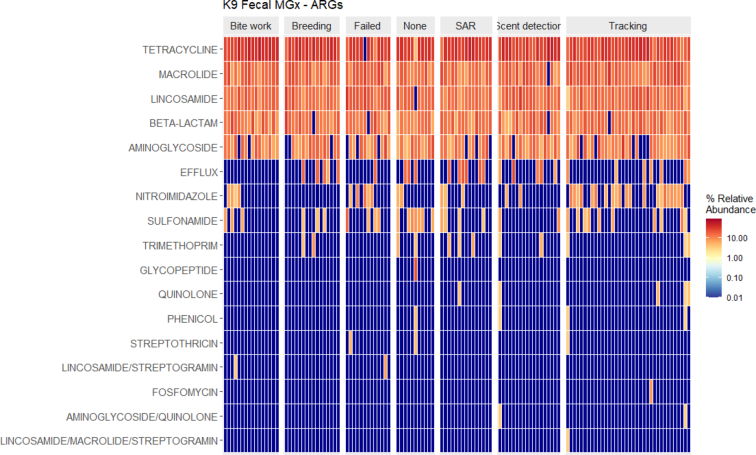
Demographic associations with presence of select ARGs within Israeli working canines.

We identified 95 ARGs in the 126 dogs from the Israeli cohort. There was a median of 14 (IQR: 6) ARGs identified in each dog (Mean: 15.2 genes, Range: 6-32 genes). The total number of ARGs did not vary much by job type (Supplemental Fig. 12). Beta diversity differed significantly by job type (See Supplemental Results including Supplemental Fig. 13). Alpha diversity differed solely on reported recent GI issues (Supplemental Fig. 14). ARGs identified in the dogs were related to 18 antimicrobial classes, but only 8 antimicrobial classes had abundance >1% and these 8 classes accounted for 98.7% of all antimicrobial resistance. Similar to the overall study population, tetracycline genes were the most dominant (33.2% relative abundance) across all dogs in the cohort. Macrolide (17.1%), lincosamide (16.4%), beta-lactam (14.1%), and aminoglycoside (10.9%) genes were also abundant in all dogs. While generally this was observed across working dog groups, some trends were observed, for example nitroimidazole resistant genes were generally more abundant among tracking dogs than other working groups (Fig. 3).

### 3.5 Analysis of specific antimicrobial resistance genes (ARGs)

When considering specific genes, among the beta lactamases ARGs from the *cfxA* family (82.2% of dogs, including variants *cfxA3, cfxA4, cfxA5,* and *cfxA6*), members of the *bla*OXA family, both potential carbapenemase (4.0% of dogs, including *bla*OXA-181 in two dogs, both from Israel, and *bla*OXA-347 in nine Israeli dogs) and non-carbapenemase (33.0% of dogs, including unclassified *bla*OXA and variants *bla*OXA-1*, bla*OXA-10*,* and *bla*OXA-184), and *bla*TEM (29.1% of dogs, including *bla*TEM-1*, bla*TEM-116*,* and *bla*TEM-135) were commonly observed. Less commonly observed was the important ESBL gene *bla*CTX-M (5.4% of dogs, specifically *bla*CTX-M-15 and *bla*CTX-M-27), ESBL gene *bla*VEB (3.6% of dogs, variants *bla*VEB-5 and *bla*VEB-6), and the ampC gene *bla*CMY (*bla*CMY-2 and *bla*CMY-4) (16 dogs, or 5.6%). Among clinically-relevant but infrequently observed genes, a colistin resistance gene, *mcr-10*, was observed in two shelter dogs from India, and *bla*SHV was observed in one shelter dog from India. *bla*NDM, *bla*VIM*, bla*KPC*,* and *bla*IMP genes were not observed. Furthermore, regarding less-prevalent ESBLs, 10 out of the 14 dogs with a *bla*CTX-M gene and all 10 with a *bla*VEB gene were from India. While 12 out of the 16 dogs carrying *bla*CMY genes were street and shelter dogs from India, the two *bla*CMY-2 carrying dogs were from Israel and South Africa, respectively.

Among aminoglycoside resistance genes, the most commonly observed were *aadE* (67.3%), *aph(3″)-1b* (36.4%), and *aph(6)-1d* (37.8%). Among macrolide resistance genes, the most commonly observed were *mef(A)/msr(D)* (89.8%) and *mef(En2)* (89.1%); *erm* genes were not among the most commonly observed. For example, *ermB* was observed in 25.9% of dogs. Among sulfonamide resistance genes, *Sul2* was most commonly observed (36.0% of dogs), whereas other clinically relevant genes were less-commonly observed (*Sul1*, 17.1%; *Sul3*, 6.2%). Among lincosamide resistance genes, the most commonly observed were *lnuAN2* (90.5%), *lnuC* (97.8%), and *lnuP* (43.6%). Among the most commonly identified genes, the most commonly identified class was the tetracycline class. The most commonly observed genes were *tet32* (47.3%), t*et(40)* (78.2%), *tet(M)* (49.8%), *tet(O)* (92.7%), *tet(Q)* (81.1%), *tet(W)* (83.3%), *tetA(P)* (71.6%), and *tetB(P)* (72.0%). Fluoroquinolone and vancomycin resistance genes were notably uncommon, with the most common *qnr* gene (*qnrS1*) observed in 14 (5.0%) dogs (10 from India and 4 from Israel). Vancomycin resistance genes (including *vanC, vanR-C, vanS-C, vanT-C*, and *vanXY-C*) were observed in no more than five dogs (1.7%) each and these genes were exclusively observed in dogs from India and Israel. No *vanA/B* genes were detected.

### 3.6 Resistome analysis - differential abundance

Differential abundance was determined by Random Forest to identify ARG abundance differences across and among groups. Many ARGs, including aminoglycoside class (e.g. *aac(6′)-lm*, *aah(2″)-lla*) were found to be differentially abundant by country of origin (Supplemental Fig. 15). The presence or absence of these ARGs could be the drivers of the difference in beta diversity that was observed among these populations. More data on resistome alpha and beta diversity among geographic cohorts as well as within different demographic groups are presented in Supplemental Materials. Among demographic groups within the Israeli canines (Supplemental Fig. 16) several ARGs (e.g. *aadS* and *mef(En2)*) had greater relative abundance in dogs with recurrent GI issues, whereas *tetA(P)* was more abundant in the Israeli dog population without recurrent GI issues. Several ARGs were also differentially abundant by job type and sterilization status (see Supplementary Results, inc. Supplemental Fig. 16).

### 3.7 Microbiome and resistome correlation - global sample

**Fig. 4:**
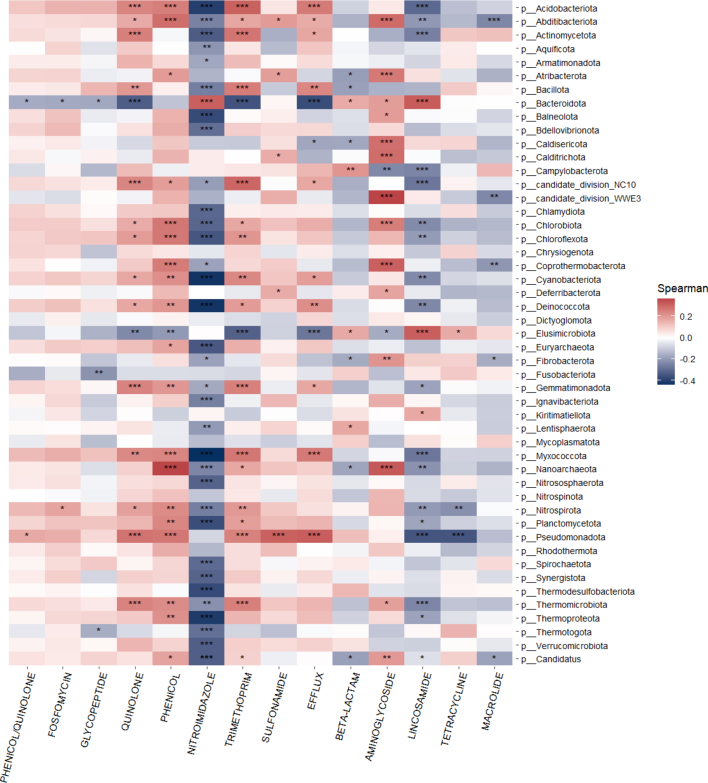

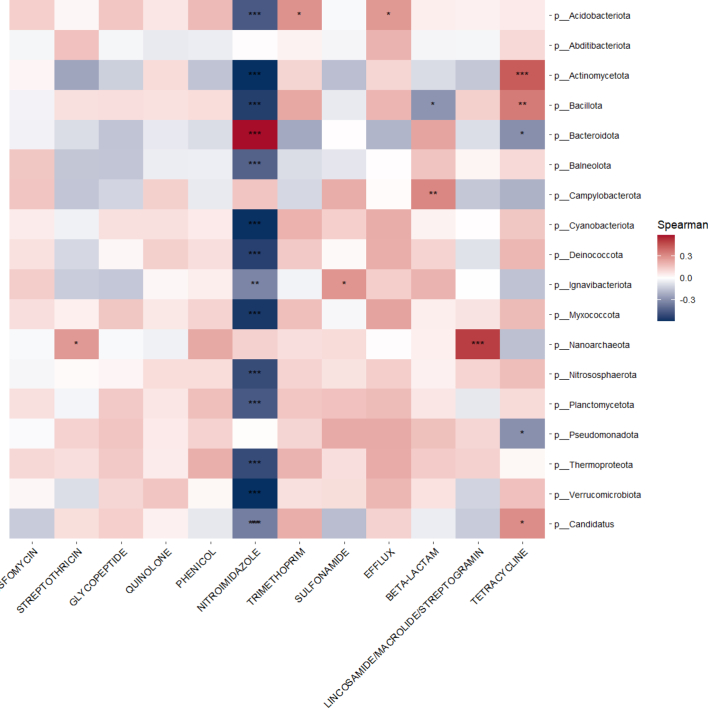
**Panel A.** Heat map of ARGs correlated with Phyla. * *P* < 0.05, ** *P* < 0.01, *** *P* < 0.001. **Panel B.** Heat map of ARGs in the Israeli dog cohort correlated with Phyla. * P < 0.05, ** P < 0.01, *** P < 0.001.

To determine whether the presence of ARGs was associated with the presence of particular bacterial taxa, we used the Spearman correlation to examine associations between the microbiome and resistome (Fig. 4, Panel A). We observed that nitroimidazole class ARGs were significantly associated, usually negatively, with a number of different bacterial phyla and that *Pseudomonadota* and *Bacteroidota* were significantly associated with a number of ARGs. Among Israeli samples specifically (Fig. 4, Panel B), nitroimidazole resistance genes were significantly associated, mostly negatively, with a number of bacterial phyla and *Nanoarchaeota* was significantly positively associated with streptothricin and lincosamide/macrolide/streptogramin class ARGs.

### 3.8 Plasmid-associated contigs

When plasmid-associated contigs were investigated, 199 unique predicted plasmids (Supplemental Sheet 1) were identified in 237 dogs (82.9%). These plasmids were highly diversified across dogs; 178 of these plasmids were identified in fewer than 10% of dogs. Some differences were observed in plasmid distribution across countries; for example *IncQ1_1* was overrepresented in canines from the US, and *IncFIC(FII)_1* was overrepresented in dogs from Laos (Supplemental Table 7). Numerous plasmids types reported to be associated with AMR were found, including *pDOp1* (77.6% of dogs), which has been noted to carry erythromycin resistance genes [18] and *colRNAI* (37.6% of dogs) which is capable of carbapenemase gene carriage [19]. Others, such as the *pBI143* plasmid (26.6% of dogs) is not associated with resistance but is often ubiquitous in human gut metagenomes [20]. The *lnc* family of plasmids, which is frequently associated with ESBL genes [21], was also frequently detected, including *lncFIB* (21.5% of dogs) and *lncFII* (20.3% of dogs). When grouping the *lnc* family of plasmids (Supplemental Table 8), *lncF* plasmids were most frequently detected (60.0% of *lnc* detects), followed by *lncQ* (11.7% of *lnc* detects). Notably, several of the *lnc* types detected (*IncF, IncI, IncH,* and *IncN*) are known to contain a variety of ARGs, while other similarly key *lnc* plasmids (i.e. *IncA/C, IncL*) were not observed [21].

### 3.9 Metagenome assembled genomes and associated resistance profiles

**Fig. 5:**
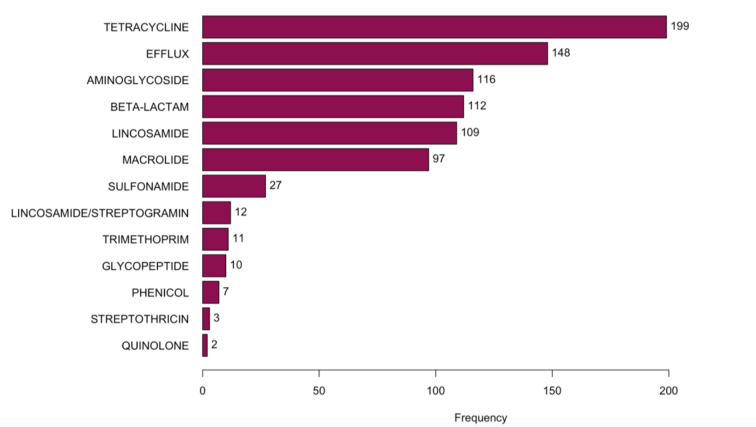
Distribution of antimicrobial resistance genes (ARGs) on metagenome assembled genomes (MAGs) from global canine metagenomes.

**Fig. 6:**
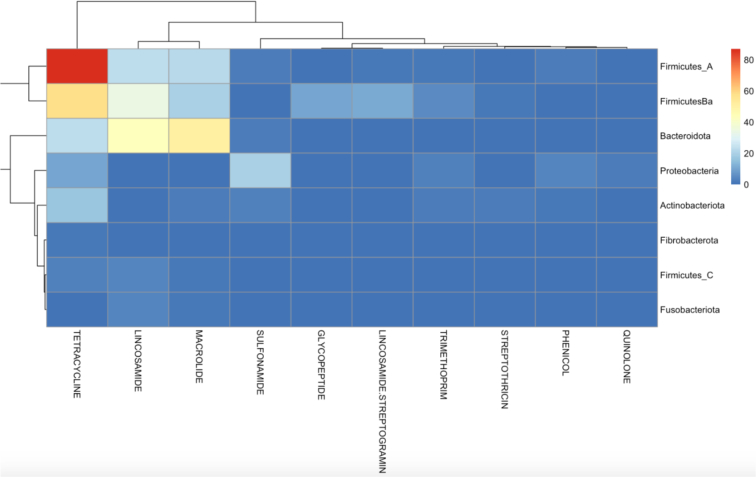
Antimicrobial resistance genes observed on Metagenome assembled genomes (MAGs) identified in global canine samples, by antimicrobial class.

A total of 10,264 MAGs were identified. 478 of these MAGs were found to contain ARGs, and among these, 848 ARGs were identified in the recovered MAGs. Among ARGs identified on MAGs, these were most commonly tetracycline genes (23.7%), efflux pumps (17.6%), aminoglycosides (13.8%), beta-lactams (13.3%), and lincosamides (13.0%) (Fig. 5, MAGs by subclass are described in Supplemental Fig. 17). These ARGs were not evenly distributed among MAGs, however. For example, efflux pumps were mostly associated with *Proteobacteria*, whereas tetracycline genes were associated with *Firmicutes* and lincosamides and macrolides were more frequently associated with *Bacteroides* (Fig. 6, Supplemental Fig. 18). Of note, Israel was under-represented in the MAG database, potentially due to the relative depth of sequencing data (Supplemental Table 9).

Of the 112 MAGs associated with the Beta-lactam class ARGs, one of these MAGs (identified as *E. coli*) was associated with a carbapenamase gene (*bla*OXA-181), and two other *E. coli* MAGs were associated with a *bla*CTX-M gene and a *bla*CTX-M-15 gene; 55 (49.1%) of these were *E. coli* MAGs associated with the *blaEC* gene. The other major group of beta-lactamase-associated MAGs were *cfxA* genes (*cfxA, cfxA3, cfxA5,* and *cfxA6*, 28 MAGs, 25%), however this was more diverse in terms of taxonomic assignments (Table 1). No MAGs were associated with colistin resistance genes.

**Table 1:** Beta-lactamase genes, and associated assigned bacterial species, observed among the MAG dataset.

Beta-Lactamase ARG	Number	%	Species identified
Carbapenemases			
*bla*OXA-181	1	0.9	*Escherichia coli*
ESBLs			
*bla*CTX-M	2	1.8	*Escherichia coli*
*bla*OXA	5	4.5	*Fusobacterium* spp.
*bla*OXA-10	1	0.9	*Providencia alcalifaciens*
*bla*OXA-85	1	0.9	*Peptostreptococcus russellii*
*bla*VEB-5	1	0.9	*Anaerobiospirillum succiniciproducens*
Beta-Lactamases			
*bla*ACI	2	1.8	*Veillonella* sp.*, Megasphaera elsdenii*
*bla*CARB-2	1	0.9	*Anaerobiospirillum succiniciproducens*
*bla*CMY	3	2.7	*Fournierella massiliensis, Escherichia coli,* *Sutterella wadsworthensis*
*bla*EC	55	49.1	*Escherichia coli*
*bla*TEM-1	6	5.4	*Escherichia coli (*5*), Allobaculum* sp. *(*1*)*
*cblA*	3	2.7	*Bacteroides uniformis*
*cepA*	3	2.7	*Bacteroides fragilis*
*cfxA*	28	25.0	*Alloprevotella sp000437675 (*2*), Amulumruptor sp900539915 (*1*), Bacteroides* sp. (1), *Bacteroides sp900766005* (1), *Muribaculaceae UBA7173 sp001701135* (1)*, Parabacteroides* sp. (1)*, Phocaeicola coprophilus* (5)*, Phocaeicola plebeius (*1)*, Phocaeicola sp900546645* (3), *Phocaeicola sp900544075* (2)*, Phocaeicola sp900546645* (2)*, Phocaeicola sp900546645 (*1*), Prevotella copri* (6*), Prevotella sp004554665 (*1*)*
**TOTAL**	**112**		

## 4 Discussion

This study presents a global overview of ARGs in domestic canine populations with a focus on working dogs, and overall we observe that there are differences in both the microbiome and resistome of canines that are connected with origin, microbiome features, and other demographic factors. Such associations are expected since prior studies [22], [23], [24], [25] have noted that canine microbiome can vary on features such as breed, age, sex, and GI disease, and human studies have noted that relationships have been observed between microbiome and resistome compositions [26]. This study expands previous research on the topic by utilizing metagenomic data and a random forest analysis which allows the observation of specific genes and bacterial groups driving the differences among groups, and the analysis of MAGs to associate bacterial genomes with ARGs.

This study demonstrates that ARG carriage in domestic canines is strongly associated with the composition of the gut microbiome, highlighting the influence of specific bacterial phyla on the resistome profile. We observed that the most abundant phyla among sampled canines were *Actinomycetota*, *Pseudomonadota*, *Bacteroidota*, *Bacillota*, and *Campylobacterota*, and furthermore, we observed distinct associations between these phyla and key ARG classes. Quinolone and trimethoprim resistance genes were strongly associated with *Actinomycetota* and *Pseudomonadota* abundance, both phyla known to harbour diverse resistomes with high rates of horizontal gene transfer [27], [28]. *Campylobacterota*, notably present in South African dogs, was specifically associated with beta-lactam resistance genes, in line with prior findings that *Campylobacter* species acquire beta-lactam resistance via environmental exposure and dietary sources, particularly poultry [29], [30]. Among commensal phyla, *Bacteroidota* and *Bacillota* are known reservoirs of resistance and mobile genetic elements (MGEs) [28], [31]. In this study we found *Bacteroidota* to be significantly associated with nitroimidazole and lincosamide resistance genes and *Bacillota* associated with quinolone and trimethoprim resistance genes. The associations reported here reinforce the role of microbiome composition in modulating ARG burden and transmission potential, validating recent studies of microbial communities and resistomes across One Health settings. By linking specific phyla to particular ARG classes observed in our dataset, we provide mechanistic insight into how gut microbiome composition may drive the risk of antimicrobial resistance gene acquisition and spread in domestic canines.

A key finding of this study were genes of potential human and veterinary public health importance. Other *bla*OXA genes have been associated with companion animal ESBL outbreaks, including *bla*OXA-181 (observed in two Israeli dogs in our study) and *bla*CMY-42 (observed in one Israeli dog and one South African dog in our study) in Switzerland; the *bla*OXA-181 gene in the Swiss study was associated with both *lncI* and *lncX3* plasmids, both of which were observed in this study [32]. *bla*OXA-181, alongside *bla*OXA-347, are two potential carbapenemases both observed exclusively in Israeli dogs in this study. A colistin resistance gene (*mcr-10*), was observed in two shelter dogs from India. Regarding colistin resistance, a 2023 paper from Japan noted observed *mcr*-harboring isolate from a dog [33] the *mcr-1* gene specifically in two dogs imported from Russia [34]. Another study done exclusively in Romanian shelter dogs had similar findings [35]. While it was expected that dogs with higher levels of exposure to waste, like street and shelter dogs, would potentially carry more significant ARGs, it is of note that the two observed potential carbapenemases were observed in dogs with controlled diets and high levels of care.

Regarding MAGs, Cusco et al. looked at MAGs from canine samples using long-read sequencing. Most of their MAG-associated ARGs were tetracyclines, followed by lincosamides and then macrolides. This agrees with our findings, with the main difference in that we observed more MAG-associated beta lactam than macrolide resistance. This, however, is not surprising considering the study material used by Cusco et al. came from a single healthy dog. This study also investigated plasmids within MAGs based on long-read sequencing, which was not possible in the current study which only included short-read datasets [36], [37]. Zhao et al. also investigated MAGs in dogs and their owners. They also noted that the most frequent ARG types were macrolides, tetracyclines, beta-lactams, and aminoglycosides, and most of their ARG-hosting MAGs belonged to *Enterobacteriaceae* [38].

### 4.1 Future areas of research and limitations

Future research should focus on transmission dynamics of ARGS between working dogs and their handlers, as Zhao et al. noted that privately-owned dogs shared resistome features with their owners. This association is not studied in terms of working dogs, either with their handlers or with their environments, ergo future research could investigate this as a uniquely paired resistome, both cross-sectionally and longitudinally [38]. Transmission dynamics among working dogs that also interface with livestock may also warrant inquiry [39]. As the majority of AMR studies in dogs have focused on isolate analysis, further metagenomic work is needed. Methodologically, long-read studies would be needed to do in-depth studies of plasmids and other mobile genetic elements. Due to the nature of using publicly accessible metagenomes, there were limitations in regard to matching sample sizes from the various origins as well as depth of the metadata. Furthermore, this study compared fecal microbiomes. Urinary or saliva microbiomes may provide different results.

## 5 Conclusions

Overall, the findings of this study suggest that canines as an AMR source within the One Health paradigm will vary widely based on location as well as exposures of the individual canine. For example, resistomes were observed to vary not just with country of origin, but also with gastrointestinal disease state, canine job type, and microbiome composition. Genes of concern such as ESBL genes, potential carbapenemases, and colistin resistance genes were comparatively infrequently observed, however plasmids associated with ESBL genes were frequently detected, suggesting the capacity for these genes to be transmitted easily when and if they are introduced to individual canines. Genes of concern were identified across the care continuum, from Indian street dogs to highly-regulated Israeli working dogs. These findings present the need to investigate diverse populations of dogs globally to better understand AMR risk from human-canine contact.

## CRediT authorship contribution statement

**Hillary A. Craddock:** Writing – review & editing, Writing – original draft, Visualization, Investigation, Formal analysis. **Yair Motro:** Writing – review & editing, Writing – original draft, Visualization, Methodology, Formal analysis, Data curation. **Katherine M. Winner:** Writing – review & editing, Writing – original draft, Visualization, Methodology, Formal analysis. **Yuval Lotem-Michaeli:** Writing – review & editing, Conceptualization. **Eran Segal:** Writing – review & editing, Methodology, Conceptualization. **Anastasia Godneva:** Writing – review & editing, Conceptualization. **Dan Grinstein:** Writing – review & editing, Conceptualization. **Jacob Moran-Gilad:** Writing – review & editing, Supervision, Project administration, Methodology, Investigation, Funding acquisition, Conceptualization.

## Declaration of competing interest

There are no conflicts of interest to disclose.

## Data Availability

Data are publicly accessible or will be made public upon publication

## Glossary

AMR: Antimicrobial resistance
ARG: Antimicrobial resistance gene
ESBL: Extended-spectrum beta-lactamase
MAG: Metagenome assembled genome
MGE: Mobile Genetic Element

## Appendix A Supplementary data

Supplementary material 1 Image 1

Supplementary material 2 Image 2

## References

[1] Kim Y. (2020). Antibiotic resistance gene sharing networks and the effect of dietary nutritional content on the canine and feline gut resistome. Anim. Microbiome.

[2] Røken M. (2022). Antimicrobial resistance—Do we share more than companionship with our dogs?. J. Appl. Microbiol..

[3] Kamathewatta K.I. (2019). Exploration of antibiotic resistance risks in a veterinary teaching hospital with Oxford Nanopore long read sequencing. PLoS One.

[4] Shnaiderman-Torban A. (2020). Extended-spectrum β-lactamase-producing enterobacterales shedding by dogs and cats hospitalized in an emergency and critical care department of a veterinary teaching hospital. Antibiotics.

[5] Shnaiderman-Torban A. (2022). Prevalence and molecular characterization of extended-spectrum β-lactamase producing enterobacterales in healthy community dogs in Israel. Antibiotics.

[6] van den Bunt G. (2020). Faecal carriage, risk factors, acquisition and persistence of ESBL-producing Enterobacteriaceae in dogs and cats and co-carriage with humans belonging to the same household. J. Antimicrob. Chemother..

[7] Haenni M. (2022). Enterobacterales high-risk clones and plasmids spreading blaESBL/AmpC and blaOXA-48 genes within and between hospitalized dogs and their environment. J. Antimicrob. Chemother..

[8] Menard J. (2022). Effect of antimicrobial administration on fecal microbiota of critically ill dogs: dynamics of antimicrobial resistance over time. Anim. Microbiome.

[9] Marchetti L., Buldain D., Gortari Castillo L., Buchamer A., Chirino-Trejo M., Mestorino N. (2021). Pet and stray dogs as reservoirs of antimicrobial-resistant *Escherichia coli*. Int. J. Microbiol..

[10] Craddock H.A. (2022). Phenotypic correlates of the working dog microbiome. NPJ Biofilms Microbiomes.

[11] Coelho L.P. (2018). Similarity of the dog and human gut microbiomes in gene content and response to diet. Microbiome.

[12] Yarlagadda K. (2022). Geographically diverse canid sampling provides novel insights into pre-industrial microbiomes. Proc. R. Soc. B Biol. Sci..

[13] R Core Team, “R: The R Project for Statistical Computing.” 2025. Accessed: Aug. 07, 2025. [Online]. Available:https://www.r-project.org/.

[14] Wickham H. (2019). Welcome to the Tidyverse. J. Open Source Softw..

[15] Wickham H. (2016).

[16] Oksanen J. (2025). vegan: Community Ecology Package. https://cran.r-project.org/web/packages/vegan/index.html.

[17] Liu C., Cui Y., Li X., Yao M. (2021). microeco: an R package for data mining in microbial community ecology. FEMS Microbiol. Ecol..

[18] Ekwanzala M.D., Dewar J.B., Kamika I., Momba M.N.B. (2020). Comparative genomics of vancomycin-resistant Enterococcus spp. revealed common resistome determinants from hospital wastewater to aquatic environments. Sci. Total Environ..

[19] Sugita K. (2021). Molecular Analysis of blaKPC-2-Harboring Plasmids: Tn4401a Interplasmid Transposition and Tn4401a-Carrying ColRNAI Plasmid Mobilization from *Klebsiella pneumoniae* to Citrobacter europaeus and *Morganella morganii* in a Single Patient. mSphere.

[20] Fogarty E.C. (2023). A highly conserved and globally prevalent cryptic plasmid is among the most numerous mobile genetic elements in the human gut. bioRxiv.

[21] Rozwandowicz M. (2018). Plasmids carrying antimicrobial resistance genes in Enterobacteriaceae. J. Antimicrob. Chemother..

[22] Deng P., Swanson K.S. (2015). COMPANION ANIMALS SYMPOSIUM: future aspects and perceptions of companion animal nutrition and sustainability. J. Anim. Sci..

[23] Deng P., Swanson K.S. (2015). Gut microbiota of humans, dogs and cats: current knowledge and future opportunities and challenges. Br. J. Nutr..

[24] Gagné J.W. (2013). Effects of a synbiotic on fecal quality, short-chain fatty acid concentrations, and the microbiome of healthy sled dogs. BMC Vet. Res..

[25] Minamoto Y. (2015). Alteration of the fecal microbiota and serum metabolite profiles in dogs with idiopathic inflammatory bowel disease. Gut Microbes.

[26] Van Gompel L. (2020). Description and determinants of the faecal resistome and microbiome of farmers and slaughterhouse workers: a metagenome-wide cross-sectional study. Environ. Int..

[27] Qiao W., Wang L., Luo Y., Miao J. (2021). Outer membrane vesicles mediated horizontal transfer of an aerobic denitrification gene between *Escherichia coli*. Biodegradation.

[28] Wang C. (2025). Global landscape of antibiotic resistance genes in the human gut microbiome metagenome-assembled genomes. BMC Microbiol..

[29] E. F. S. Authority and European Centre for Disease Prevention and Control (2017). The European Union summary report on trends and sources of zoonoses, zoonotic agents and food-borne outbreaks in 2016. EFSA J..

[30] Casagrande Proietti P. (2020). Beta-lactam resistance in *Campylobacter coli* and *Campylobacter jejuni* chicken isolates and the association between blaOXA-61 gene expression and the action of β-lactamase inhibitors. Vet. Microbiol..

[31] Gillieatt B.F., Coleman N.V. (2024). Unravelling the mechanisms of antibiotic and heavy metal resistance co-selection in environmental bacteria. FEMS Microbiol. Rev..

[32] Nigg A. (2019). Shedding of OXA-181 carbapenemase-producing *Escherichia coli* from companion animals after hospitalisation in Switzerland: an outbreak in 2018. Eurosurveillance.

[33] Yasugi M. (2023). Genetic and phenotypic analyses of mcr-harboring extended-spectrum β-lactamase-producing *Escherichia coli* isolates from companion dogs and cats in Japan. Vet. Microbiol..

[34] Johansson V., Nykäsenoja S., Myllyniemi A.-L., Rossow H., Heikinheimo A. (2022). Genomic characterization of ESBL/AmpC-producing and high-risk clonal lineages of *Escherichia coli* and *Klebsiella pneumoniae* in imported dogs with shelter and stray background. J. Glob. Antimicrob. Resist..

[35] Cozma A. (2019). Characterisation of extended β-lactamases and plasmid mediated quinolones resistancein *Escherichia Coli* from shelter dogs. Bull. Univ. Agric. Sci. Vet. Med. Cluj-Napoca Vet. Med..

[36] Cuscó A., Pérez D., Viñes J., Fàbregas N., Francino O. (2021). Long-read metagenomics retrieves complete single-contig bacterial genomes from canine feces. BMC Genomics.

[37] Cuscó A., Pérez D., Viñes J., Fàbregas N., Francino O. (2022). Novel canine high-quality metagenome-assembled genomes, prophages and host-associated plasmids provided by long-read metagenomics together with Hi-C proximity ligation. Microb. Genomics.

[38] Zhao R. (2022). The co-occurrence of antibiotic resistance genes between dogs and their owners in families. iMeta.

[39] Cohen A. (2022). Fecal microbiome features associated with extended-spectrum β-lactamase-producing enterobacterales carriage in dairy heifers. Animals.

