# Supplementary material for: Metagenomic analysis of antimicrobial resistance genes in domestic canines
Source: One Health. 2026 Mar 4;22:101380. doi: 10.1016/j.onehlt.2026.101380 (PMC13053998; doi:10.1016/j.onehlt.2026.101380)
Supplement: Supplementary file 1 — Supplementary material 1 [file mmc1.docx]

**Supplementary Material**

**Supplemental Methods**

*Israeli Canine Sample Collection and Processing*

Dogs receiving antibiotics in the preceding four weeks were excluded. Samples were snap frozen and kept at -80 Celsius until processing. DNA was extracted using the PowerMicrobial/PowerSoil DNA kits (Qiagen, Hilden, Germany). Metadata were collected including basic demographic information (e.g. sex, sterilization status, operational job, breed) and factors which are known to influence microbiota composition in canines (antibiotic and proton pump inhibitor (PPI) administration history, diet, history of gastrointestinal disorders).

*Bioinformatics*

In total, 273 samples (126 Israeli and 147 global) underwent QC using fastp (v.0.23.2; with defaults) [1], resulting in a total of 1,650,149,786 reads. The number of reads per sample ranged from 84,820 - 40,521,884 with an average of 1,389,438 ± 9,295,226 reads.

Taxonomic profiling was performed on the QC reads using kraken2 (v.2.1.2) [2] with the refseq100GB database [3], and the output report for each sample converted to mpa format using taxonkit (v.0.15.0) [4] and collated together with the combine_mpa.py script from KrakenTools (v.1.2) [5].

ARGs profiling was performed on the QC reads using ARGs_OAP (v.3.2.3) [6]. The reads were then assembled using Megahit (v.1.2.9https://github.com/voutcn/megahit) [7]. For a subset of global samples (n=62) multiple sequencing runs were available for coassembly also using Megahit. was performed The resistome and plasmidome of the metagenome assemblies assembled MGx samplesAGs (Metagenome-assembled genomes) were predicted using Abricate (v.1.0.1) (https://github.com/tseemann/abricate), AMRFinderPlus (v.3.10.42https://github.com/ncbi/amr) [8], the CARD database [9], the MEGARes database [10], and the plasmidfinder database [11].

The metagenome assembled genomes (MAGs) were isolated using SemiBin (v.1.0.1https://github.com/BigDataBiology/SemiBin; using single_easy_bin and --environment dog_gut) [12], minimap2 (v.2.24-r1122https://github.com/lh3/minimap2) [13], and samtools (v.1.15https://github.com/samtools/samtools) [14]. The taxonomic assignments of the MAGs were determined using GTDBTk (v.2.1.0https://github.com/Ecogenomics/GTDBTk) [15]. The MAGs were quality controlled using CheckM (v.1.0.11https://github.com/Ecogenomics/CheckM) [16], GUNC (v.1.0.1-2https://github.com/grp-bork/gunc) [17], tRNAscan-SE (v.2.0.6-2https://github.com/UCSC-LoweLab/tRNAscan-SE) [18], barRNAp (v.0.9-1https://github.com/tseemann/barrnap), and prok-quality (v.1.2.3https://github.com/metashot/prok-quality) [19]. The MAGs were dereplicated using dRep (v.2.6.2-2https://github.com/MrOlm/drep) [20]. The MLST, resistome, and plasmidome of the good quality and dereplicated MAGs were determined using MLST (v.2.22.1) (https://github.com/tseemann/mlst), Abricate (https://github.com/tseemann/abricate), AMRFinderPlus (https://github.com/ncbi/amr), the CARD database (https://github.com/arpcard/amr_curation) , the MEGARes database (https://db.meglab.org/), and the plasmidfinder database (https://cge.cbs.dtu.dk/services/PlasmidFinder/).

We used the *microeco* package in R to normalize samples by total sum scaling. No OTUs were excluded from the dataset to account for background contamination. Relative abundance calculations were performed with the *microeco* package in R [21]. Diversity comparisons were performed by calculating the Shannon diversity index [22] (an integrative index of community richness and evenness) and the Bray-Curtis dissimilarity index [23] (a measure of compositional dissimilarity) using the *vegan* package in R.

**Supplemental Results**

*Supplemental Demographic information*

Demographic information was inconsistent regarding the dogs and dependent on origin. Due to the sparse nature of demographic data, only location, sex, and breed categories were compared among the global population; 51% of dogs with data on sex were male; sex did not differ by origin (p=0.1), with the standout location being India (71.0% male among dogs with data on sex). Breed categories included Belgian and Dutch Shepherd (malinois and non-malinois), Africanis, Beagle, Corgi, German Shepherd Dog (GSD), Labrador, and Other. Breed data were only available for dogs from South Africa, Israel, and the US, and breeds significantly differed by origin (P<0.0001). SA dogs were further classified by origin into farm dogs (n=2, 11.1%), suburban dogs (n=4, 22.2%) and urban dogs (n=12, 66.7%), and Indian dogs were classified into shelter dogs (n=20, 58.9%) and street dogs (n=14, 41.2%). Demographic data are summarized in Supplemental Table 1.

Supplemental Table 1: Demographics of canine population, by origin.

| **Supplemental Table 1. Demographic Characteristics** | | | | | |
| --- | --- | --- | --- | --- | --- |
|  | **Israel** | **India** | **Laos** | **South Africa** | **USA** |
|  | 126 (46.2) | 34 (12.5) | 28 (10.3) | 19 (7) | 66 (24.2) |
| **Sex** |  |  |  |  |  |
| Female | 66 (52.4) | 9 (26.5) |  | 9 (47.4) | 30 (45.5) |
| Male | 59 (46.8) | 22 (64.7) |  | 9 (47.4) | 32 (48.5) |
| NA | 1 (0.8) | 3 (8.8) |  | 1 (5.3) | 4 (6.1) |
| **Breed** |  |  |  |  |  |
| Malinois | 54 (42.9) |  |  | 0 | 0 |
| Belgian/  Dutch Shepherd | 22 (17.5) |  |  | 1 (5.3) | 0 |
| Corgi | 8 (6.3) |  |  | 0 | 0 |
| GSD | 15 (11.9) |  |  | 0 | 0 |
| Labrador | 8 (6.3) |  |  | 4 (21.1) | 29 (43.9) |
| Africanis | 0 |  |  | 2 (10.5) | 0 |
| Beagles | 0 |  |  | 0 | 37 (56.1) |
| Other | 19 (15.1) |  |  | 12 (63.2) | 0 |
| **Demographics for Israeli Canines** | | | | | |
| **Sterilized** |  |  |  |  |  |
| Yes | 15 (87.3) |  |  |  |  |
| No | 110 (11.9) |  |  |  |  |
| NA | 1 (0.8) |  |  |  |  |
| **Frequent GI Issues** |  |  |  |  |  |
| Yes | 14 (11.1) |  |  |  |  |
| No | 112 (88.9) |  |  |  |  |
| **Recent antibiotics** |  |  |  |  |  |
| Yes | 23 (18.3) |  |  |  |  |
| No | 98 (77.8) |  |  |  |  |
| NA | 5 (4) |  |  |  |  |
| **Job** |  |  |  |  |  |
| Bite work | 16 (12.7) |  |  |  |  |
| Breeding | 16 (12.7) |  |  |  |  |
| Search and Rescue | 15 (11.9) |  |  |  |  |
| Scent detection | 18 (14.3) |  |  |  |  |
| Tracking | 36 (28.6) |  |  |  |  |
| Failed | 13 (10.3) |  |  |  |  |
| NA | 12 (9.5) |  |  |  |  |
| Data are presented as n (%) | | | | | |

Supplemental Microbiome Analysis Results

We performed microbiome analysis on samples from 273 dogs. Shotgun sequencing of fecal samples resulted in a total of 1,650,149,786 sequences. The number of reads per sample ranged from 84,820 - 40,521,884 with an average of 1,389,438 ± 9,295,226 reads. Bacterial taxa varied largely in their proportions between individuals; 49 phyla were found across all samples, but only 6 phyla had abundance > 0.1% and accounted for more than 99% of relative abundance (Supplemental Figure 1 and Supplemental Table 2). The most abundant bacterial phyla were the *Bacteroidota* (50.2% ± 31.0%) and *Bacillota* (32.7% ± 23.2%).

Among them a total of 707 bacterial families were identified but only 35 had abundance > 0.1%. These families accounted for 97.69% of total abundance (Supplemental Table 3). The most abundant families were *Prevotellaceae* (33.7% ± 32.1%), *Bacteroidaceae* (14.7% ± 14.7%), and *Lachnospiraceae* (10.3% ± 11.4%). Analysis of bacterial genera revealed 3,744 genera, of which 51 had abundance > 0.1% and accounted for 94.43% of total abundance (Table C). The most abundant genera in the dogs were *Segatella* (33.1% ± 32.0%), *Phocaeicola* (11.5 ± 12.2), *Mediterraneibacter* (5.5% ± 8.0%), and *Collinsella* (4.7% ± 6.6%).

**Supplemental Table 2. Relative Abundance of Leading Phyla**

| **Phylum** | **Mean (%)** | **Min (%)** | **Max (%)** | **Median (%)** | **IQR (%)** |
| --- | --- | --- | --- | --- | --- |
| *Bacteroidota* | 50.2 | 0.1 | 96.9 | 51.9 | 58.9 |
| *Bacillota* | 32.7 | 1.9 | 93.8 | 27.8 | 37.9 |
| *Actinomycetota* | 6.7 | 0.1 | 48.4 | 3.6 | 7.7 |
| *Pseudomonadota* | 6.2 | 0.3 | 76.2 | 2.8 | 4.1 |
| *Campylobacterota* | 2.8 | 0.0 | 72.6 | 0.1 | 0.7 |
| *Fusobacteriota* | 1.1 | 0.0 | 23.5 | 0.3 | 0.6 |
| **Total** | **99.6** |  |  |  |  |

**
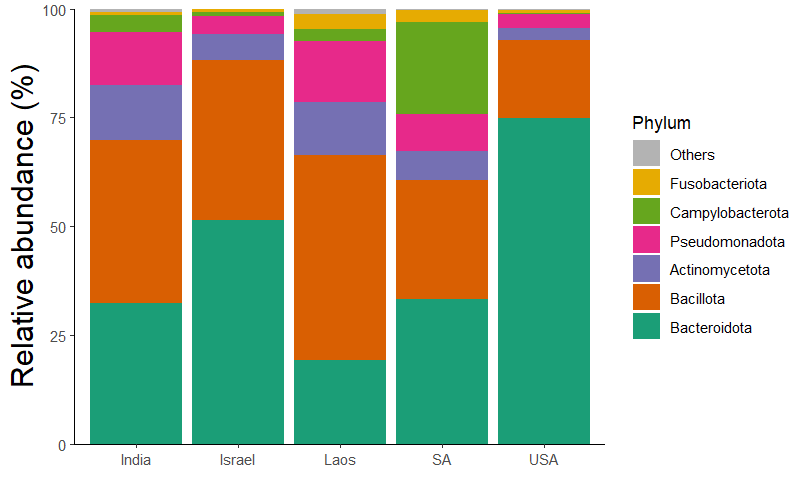
**

**Supplemental Figure 1.** Relative abundance of top 8 most abundance bacterial phyla found among studies canine samples, clustered by country of origin

**Supplemental Table 3. Relative Abundance of Leading Bacterial Families**

| **Family** | **Mean (%)** | **Min (%)** | **Max (%)** | **Median (%)** | **IQR (%)** |
| --- | --- | --- | --- | --- | --- |
| *Prevotellaceae* | 33.7 | 0.0 | 93.9 | 23.1 | 62.0 |
| *Bacteroidaceae* | 14.7 | 0.0 | 74.6 | 9.1 | 16.2 |
| *Lachnospiraceae* | 10.3 | 0.1 | 63.4 | 6.7 | 12.4 |
| *Lactobacillaceae* | 6.0 | 0.0 | 73.9 | 0.5 | 4.6 |
| *Coriobacteriaceae* | 4.7 | 0.0 | 40.2 | 2.0 | 5.9 |
| *Erysipelotrichaceae* | 4.2 | 0.0 | 33.9 | 2.1 | 4.4 |
| *Peptostreptococcaceae* | 3.4 | 0.0 | 28.3 | 1.8 | 3.4 |
| *Clostridiaceae* | 2.8 | 0.1 | 43.6 | 1.0 | 2.3 |
| *Enterobacteriaceae* | 2.6 | 0.0 | 71.8 | 0.1 | 0.7 |
| *Helicobacteraceae* | 2.2 | 0.0 | 72.3 | 0.1 | 0.4 |

**Supplemental Table 4. Relative Abundance of Leading Bacterial Genera**

| **Genus** | **Mean (%)** | **Min (%)** | **Max (%)** | **Median (%)** | **IQR (%)** |
| --- | --- | --- | --- | --- | --- |
| *Segatella* | 33.1 | 0.0 | 93.1 | 22.6 | 62.0 |
| *Phocaeicola* | 11.5 | 0.0 | 65.6 | 6.6 | 12.6 |
| *Mediterraneibacter* | 5.5 | 0.0 | 55.9 | 2.6 | 6.3 |
| *Collinsella* | 4.7 | 0.0 | 10.2 | 2.0 | 5.9 |
| *Bacteroides* | 3.2 | 0.0 | 30.8 | 1.8 | 2.5 |
| *Ligilactobacillus* | 3.2 | 0.0 | 61.2 | 0.1 | 1.4 |
| *Blautia* | 2.9 | 0.0 | 29.8 | 1.6 | 3.3 |
| *Clostridium* | 2.3 | 0.0 | 41.2 | 0.7 | 1.9 |
| *Escherichia* | 2.2 | 0.0 | 69.2 | 0.0 | 0.5 |
| *Helicobacter* | 2.2 | 0.0 | 72.4 | 0.1 | 0.1 |
| *Peptacetobacter* | 2.2 | 0.0 | 23.4 | 0.9 | 2.1 |

*Microbiome - Alpha diversity*

Alpha diversity was measured using the Shannon diversity index, a measure which takes into account the number of microbial species present (richness) and their relative abundance (evenness) within each sample. Some countries of origin were found to be significantly associated with alpha-diversity (e.g., USA has a significantly lower Shannon index than Laos, P < 0.0001, Wilcoxon Rank Sum). Of note, the dogs from India and Laos, which comprise mostly of shelter and street dogs, had a higher Shannon index, indicating higher within-sample diversity, and Israeli and US dogs, which represent working and laboratory dogs, respectively, have the lowest Shannon diversity. However Israeli versus global canines was not significantly associated with alpha-diversity (P = 0.44, Wilcoxon Rank Sum).


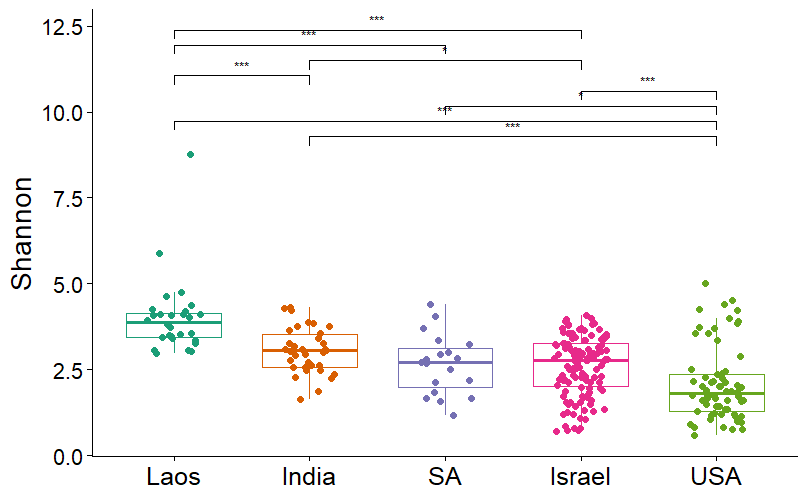


**Supplemental Figure 2A.** Alpha diversity in Shannon index by country of origin. * p<0.05, ** p<0.01, *** p<0.001


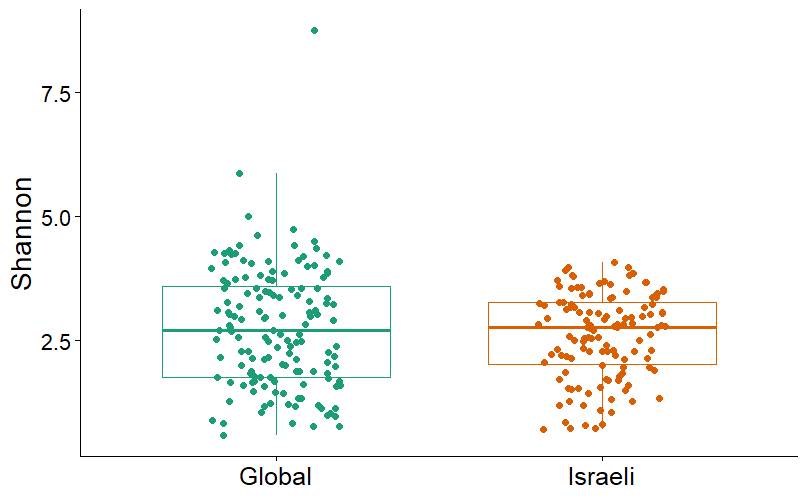


**Supplemental Figure 2B.** Alpha diversity in Shannon index between Israeli and global canines.

*Microbiome - Beta diversity analysis*

Beta diversity refers to the similarity or dissimilarity between communities, indicating the overlap in microbial composition between sample pairs. Beta diversity was compared between groups using the Bray-Curtis dissimilarity index. A significant difference in beta diversity was observed by canine country of origin (for all comparisons P≤0.02, Wilcoxon Rank Sum) and specifically between Israeli and global canines (P<0.0001, Wilcoxon Rank Sum) (Supplemental Figure 3). The significant difference in beta diversity between countries of origin indicated that canines from each country of origin have a microbial community structure that is distinct from the other countries of origin. Dissimilarity between microbial communities based on country of origin was further confirmed after performing statistical correction with PERMANOVA. Dissimilarity of all countries of origin (F = 15.59, P = 0.001, PERMANOVA) and between Israeli and global canines (F = 9.43, P = 0.001, PERMANOVA) were both significant.

**Supplemental Figure 3A.** Beta-diversity in Bray-Curtis dissimilarity index by country of origin.


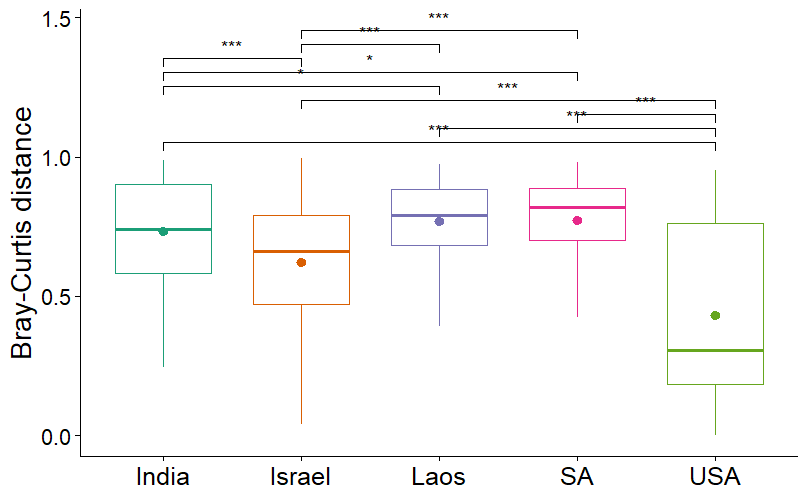

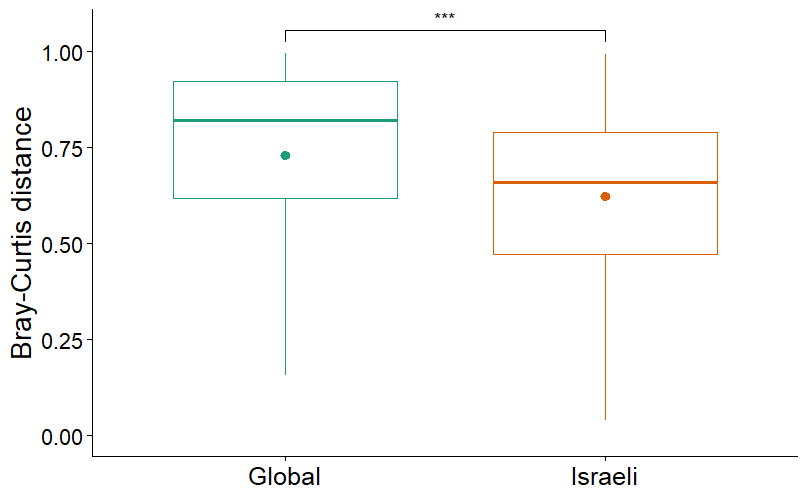


**Supplemental Figure 3B.** Beta-diversity in Bray-Curtis dissimilarity index between Israeli and Global canines. * p<0.05, ** p<0.01, *** p<0.001


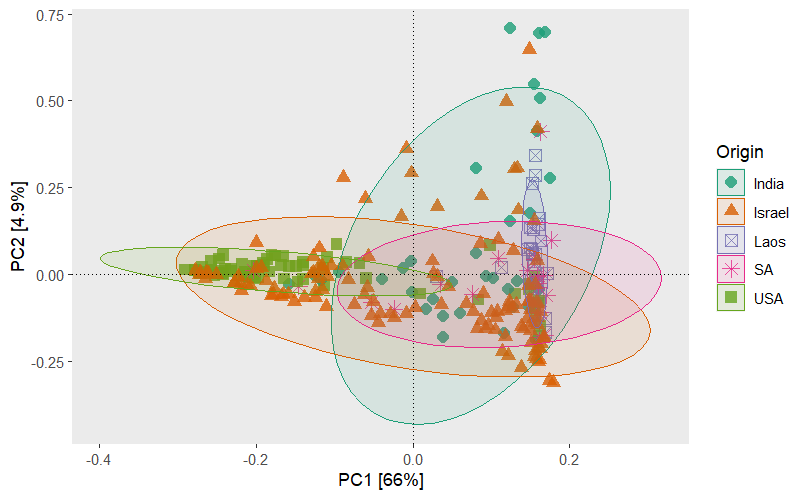


**Supplemental Figure 4.** PCA plot demonstrates a discernible difference in microbial community structure among domestic canines based on country of origin.

* SA = South Africa

*Microbiome - Differential Abundance*

Differential abundance was determined by Random Forest to identify community differences across groups. *Campylobacterota* was more abundant in the global canine population compared to the Israeli canine population (Supplemental Figure 5); this bacterial phylum could be the driver of the difference in beta diversity that was demonstrated between these groups. Interestingly, the relative abundance of *Campylobacterota* was far higher in dogs from South Africa compared to all other countries of origin, which would explain why this same phylum is differentially abundant across Israeli and global populations. Across countries of origin, the relative abundance of the phylum *Bacteroidota* was found to be highest in dogs from the USA. In contrast, the relative abundances of the phyla *Actinomycetota* and *Bacillota* were lowest in dogs from the USA compared to other countries of origin.
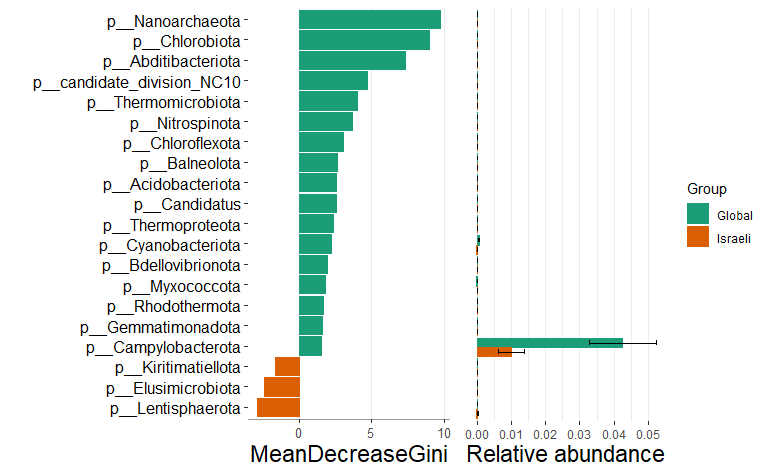


**Supplemental Figure 5A.** Random forest analysis identifies *Campylobacterota* as differentially abundant across global and Israeli canine populations.


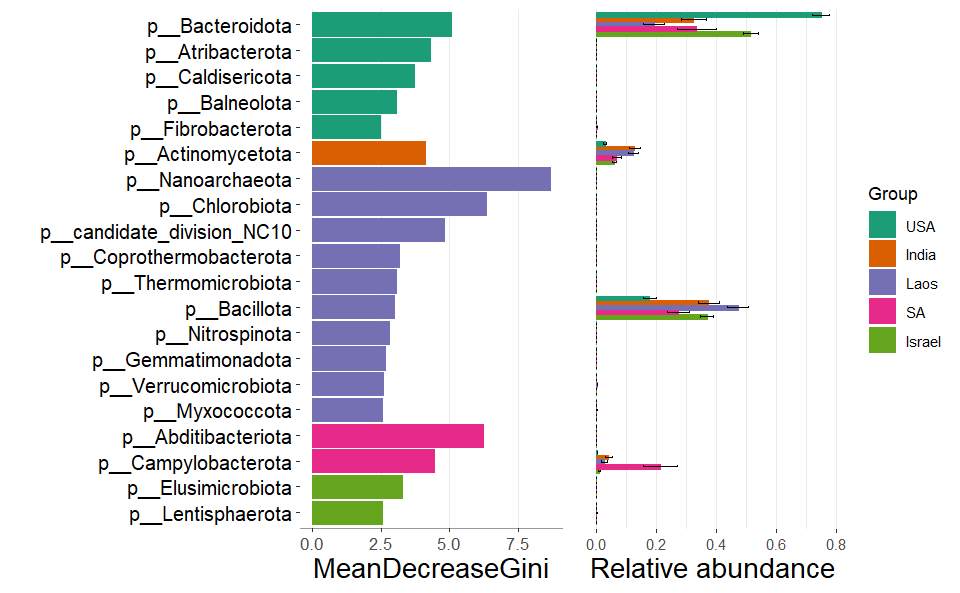


* SA = South Africa

**Supplemental Figure 5B.** Random forest analysis identifies *Bacteroidota*, *Actinomycetoma*, *Bacillota*, and *Campylobacterota* as differentially abundant across countries of origin.

*Resistome analysis*

**Supplemental Table 5. Relative Abundance of Leading ARGs**

| **ARG** | **Mean (%)** | **Median (%)** | **IQR (%)** |
| --- | --- | --- | --- |
| Inu(C) | 6.2 | 5.6 | 3.9 |
| Inu(AN2) | 5.4 | 5.0 | 3.9 |
| mef(En2) | 5.0 | 4.5 | 4.0 |
| tet(O) | 4.9 | 4.3 | 4.0 |
| mef(A) | 4.9 | 4.8 | 3.4 |
| tet(Q) | 4.5 | 4.2 | 4.4 |
| tetA(P) | 4.3 | 5.0 | 6.9 |
| tet(W) | 4.3 | 3.8 | 3.7 |
| tet(40) | 3.9 | 3.6 | 3.8 |
| cfxA6 | 3.6 | 3.2 | 5.6 |
| tetB(P) | 3.4 | 3.2 | 5.3 |
| aadE | 3.2 | 3.2 | 5.0 |
| tet(M) | 2.3 | 0.0 | 4.2 |

**Supplemental Table 6. Relative Abundance of Leading ARG Classes**

| **ARG Class** | **Mean (%)** | **Median (%)** | **IQR (%)** |
| --- | --- | --- | --- |
| Tetracycline | 31.7 | 31.8 | 11.9 |
| Macrolide | 16.0 | 15.4 | 8.9 |
| Lincosamide | 14.5 | 14.3 | 8.2 |
| Aminoglycoside | 13.9 | 13.3 | 11.7 |
| Beta-Lactam | 12.6 | 12.5 | 7.3 |
| Efflux | 4.4 | 0.0 | 8.6 |


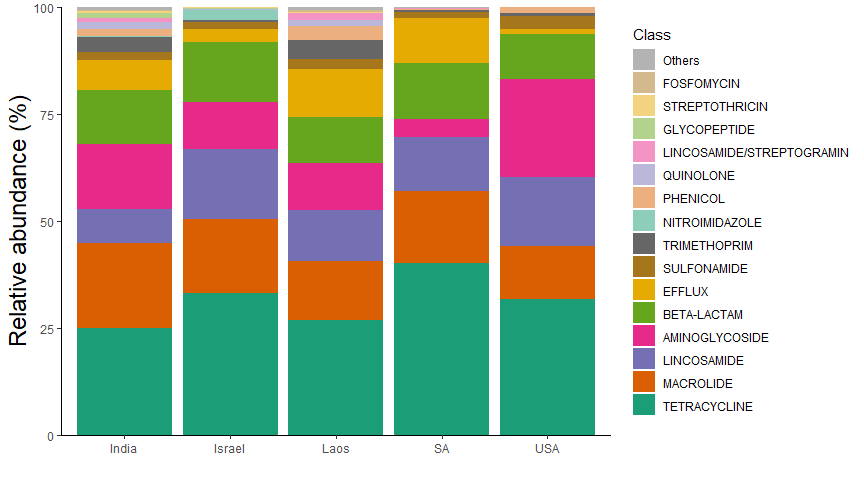


**Supplemental Figure 6.** Prevalence of antimicrobial resistance genes by drug class.


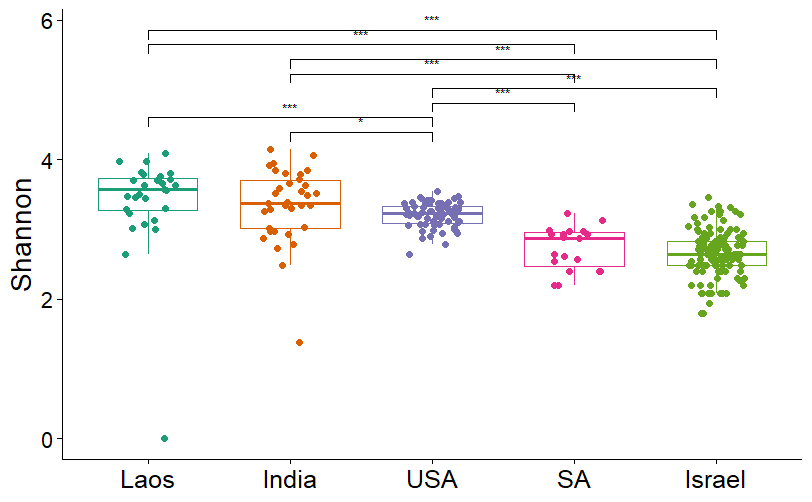


**Supplemental Figure 7.** Alpha diversity of ARGs in Shannon Index between countries of origin. * Pp<0.05, ** Pp<0.01, *** Pp<0.001

SA = South Africa


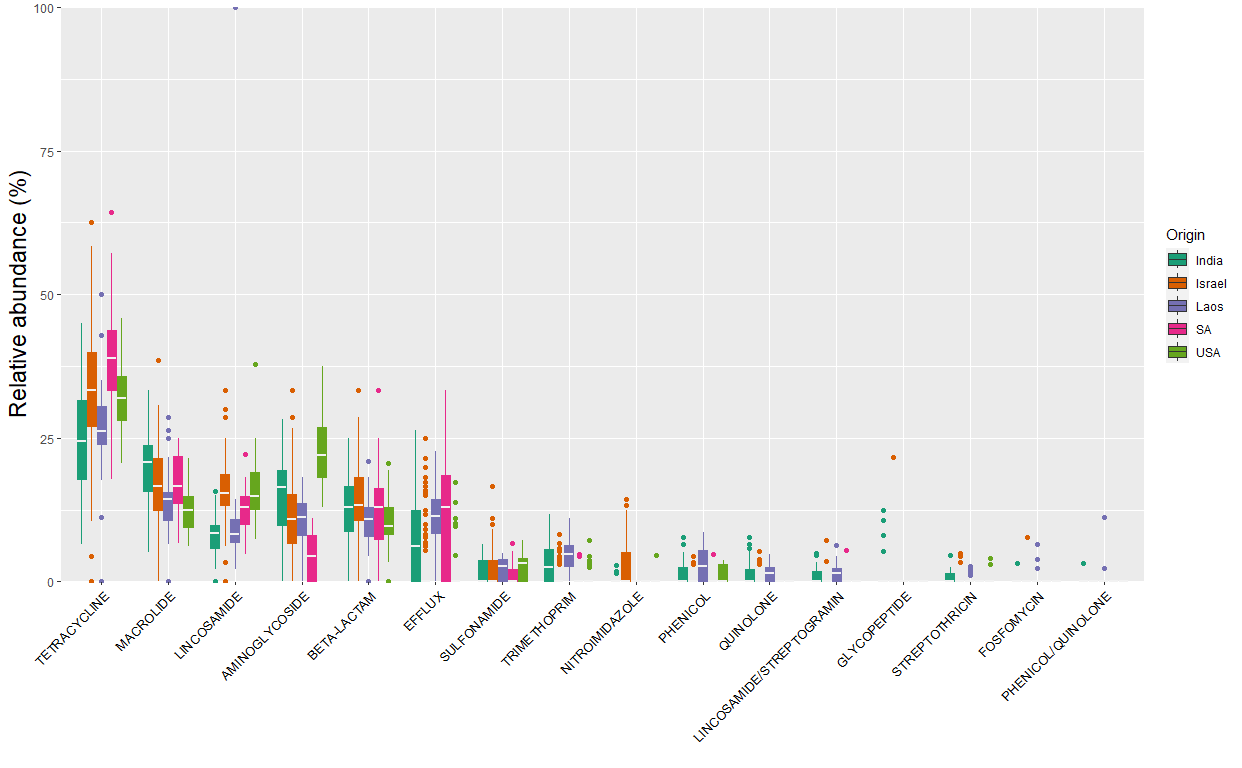


**Supplemental Figure 8.**  Relative abundance of antimicrobial resistance genes by drug class.

*Resistome analysis - Alpha diversity*

Alpha diversity of resistome features was measured using the Shannon index. All countries had significantly different resistome alpha-diversity between one another, like India-SA (P < 0.0001, Wilcoxon Rank Sum) (Supplemental Figure 7). Further, Israeli dogs had the smallest Shannon diversity index of all the countries and their alpha-diversity was significantly different from the global dog cohort (P < 0.0001, Wilcoxon Rank Sum) (Supplemental Figure 9). A similar pattern was observed regarding alpha diversity in microbiome and alpha diversity in resistomes, however of note resistome of the SA dogs had a lower Shannon index overall than the US laboratory dogs.


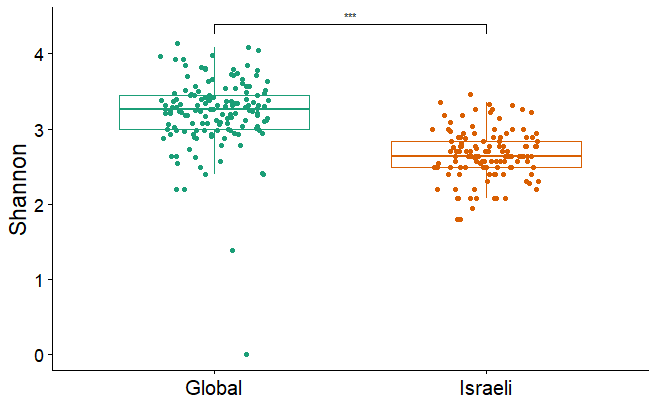


**Supplemental Figure 9.** Alpha diversity of ARGs in Shannon index between global and Israeli dogs. * p<0.05, ** p<0.01, *** p<0.001

*Resistome analysis - Beta diversity*

Beta diversity was compared among groups using the Bray-Curtis dissimilarity index. A significant difference in beta-diversity was observed between every country of origin (P < 0.01, Wilcoxon Rank Sum) (Supplemental Figure 10). A significant difference was also observed between the global and Israeli dogs (P < 0.0001, Wilcoxon Rank Sum) (Supplemental Figure 11). Dissimilarity in unique ARG presence based on country of origin was further confirmed after performing statistical correction with PERMANOVA. Dissimilarity of all countries of origin (F = 28.50, P = 0.001, PERMANOVA) and between Israeli and global canines (F = 45.68, P = 0.001, PERMANOVA) were both significant. The significant difference in beta diversity among these groups demonstrates that the number of unique ARGs in the canine samples is different depending on the country of origin. This could indicate that exposures are different and/or some feature of these groups influences canine susceptibility to acquisition of specific ARGs.


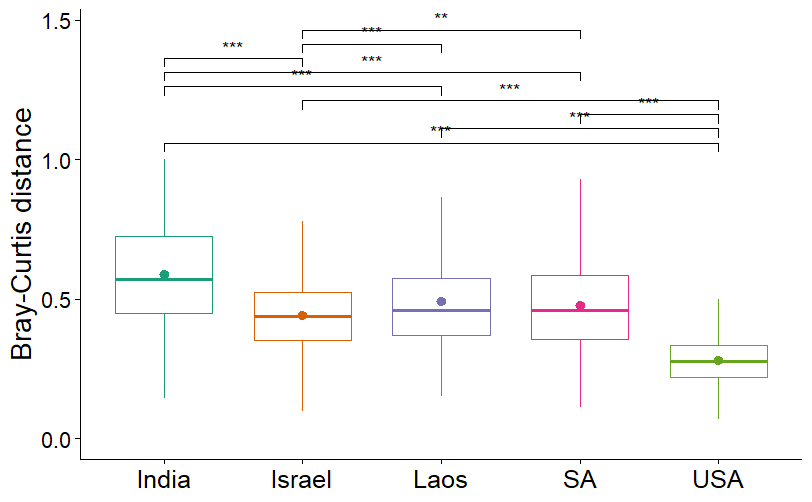

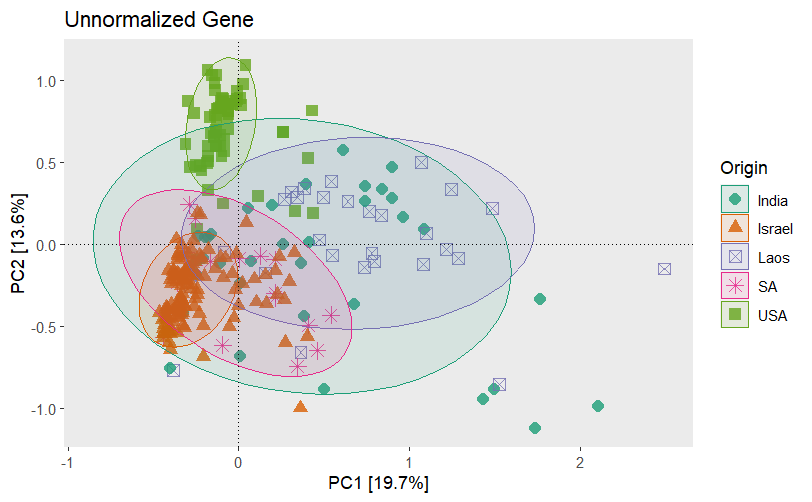


**Supplemental Figure 10.** a) Beta-diversity in Bray-Curtis dissimilarity index by country of origin. b) PCA plot demonstrates a discernible difference in ARG presence by country of origin. * p<0.05, ** p<0.01, *** p<0.001


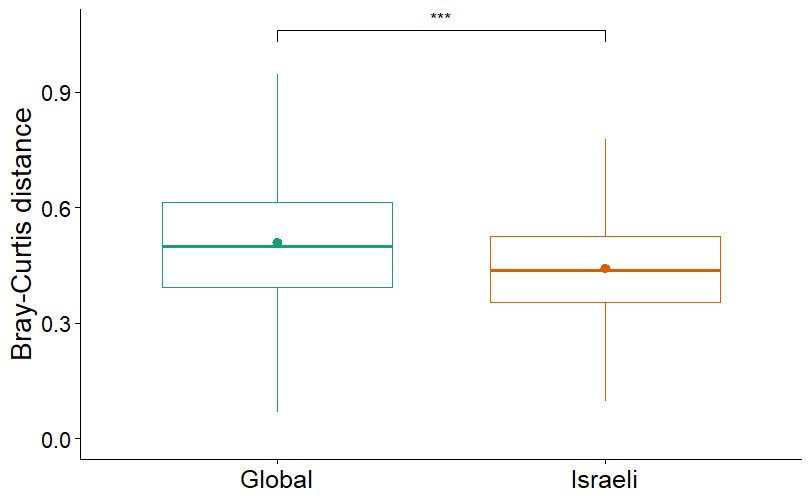

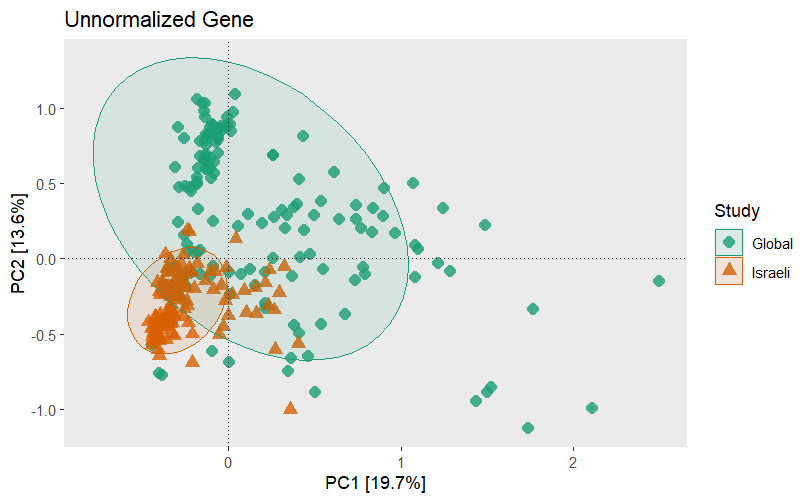


**Supplemental Figure 11.** a) Beta-diversity in Bray-Curtis dissimilarity index between global and Israeli dogs. b) PCA plot demonstrates a discernible difference in ARG presence between Israeli and global dogs. * p<0.05, ** p<0.01, *** p<0.001


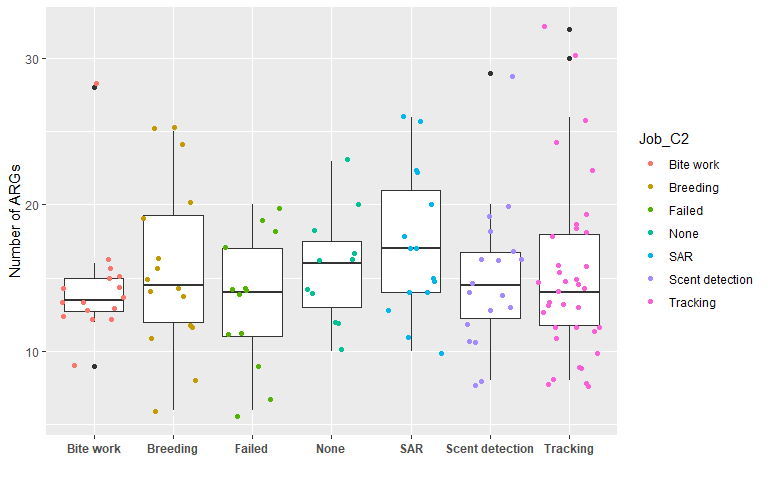


**Supplemental Figure 12.** Number of ARGs in Israeli samples by job type.

*Beta Diversity analysis of Israeli canine resistome*

Beta diversity was compared between groups using the Bray-Curtis dissimilarity index. A significant difference was observed in beta-diversity by job type (For example, P < 0.0001 for SAR vs Scent detection comparison) (Supplemental Figure 13), sex (P < 0.0001), sterilization (P < 0.0001), pack community (P < 0.0001), recent exposure to antibiotics (P = 0.0006), and recurrent GI issues (P < 0.0001) by Wilcoxon Rank Sum test. However, dissimilarity in ARG presence could not be demonstrated after performing statistical correction with PERMANOVA based on sex, pack community, or recent exposure to antibiotics. There was no significant difference in beta-diversity between adult dogs and puppies (P = 0.8, Wilcoxon Rank Sum).

Job type (F = 2.0212, P = 0.001) (Supplemental Figure 13), sterilization (F = 2.3315, P = 0.01), and recurrent GI issues (F = 2.748, P = 0.008) still demonstrated significant differences in beta diversity even after statistical correction with PERMANOVA. These differences demonstrate that the number of unique ARGs in the canine samples is different depending on job, sterilization status, and reported recurrent GI issues.

*
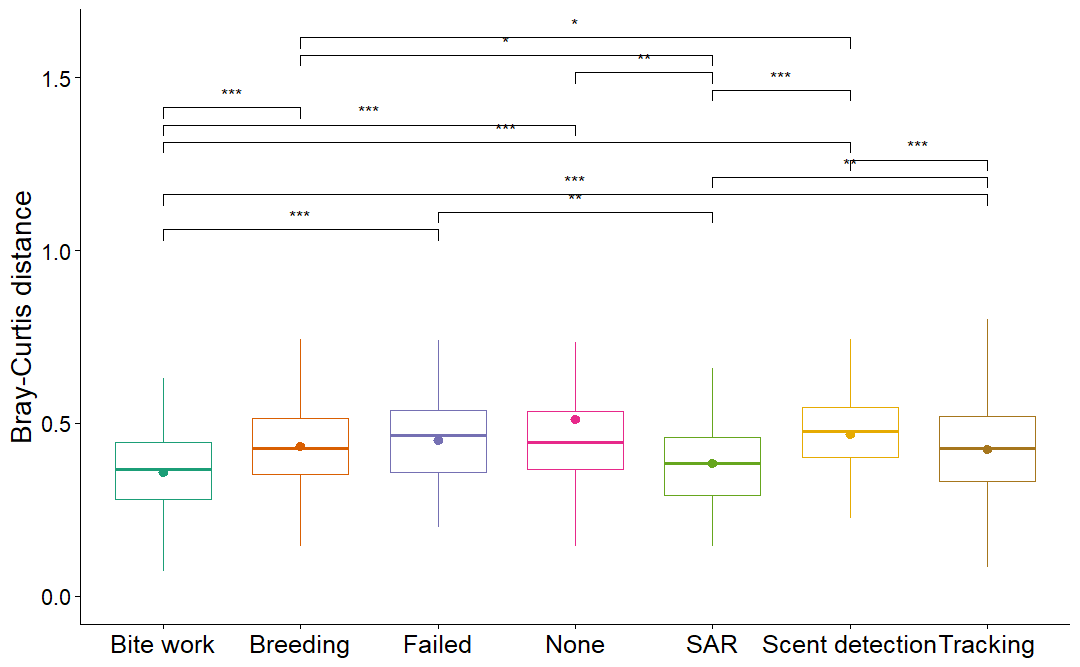

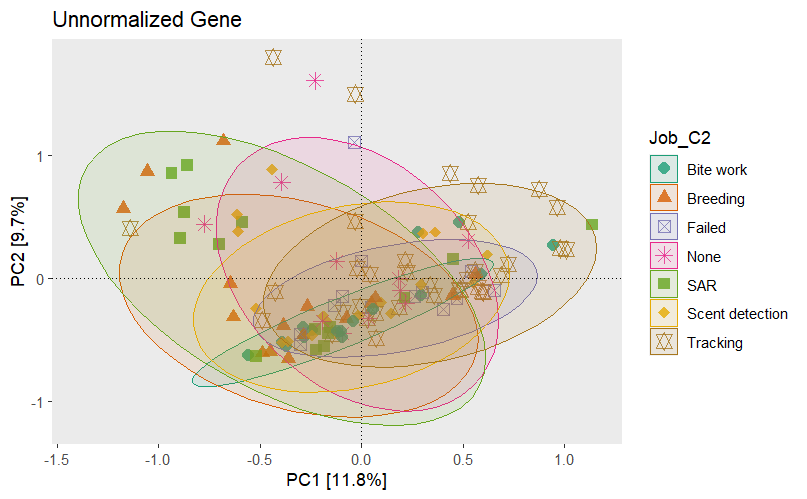
*

**Supplemental Figure 13.** a) Beta diversity in Bray-Curtis dissimilarity by job type. b) PCA plot demonstrates a discernible difference in ARG presence by job type. * p<0.05, ** p<0.01, *** p<0.001

*Alpha diversity analysis of Israeli canine resistome*

Alpha diversity of resistome features was measured by the Shannon index. Job type, sex, sterilization, age, pack community, and recent exposure to antibiotics were all not found to be significantly associated with alpha-diversity using the Wilcoxon Rank Sum test. Recurrent gastrointestinal issues were found to be significantly associated with alpha-diversity (P = 0.0126, Wilcoxon Rank Sum) (Supplemental Figure 14); dogs reported to have recurrent GI issues generally had lower alpha diversity.


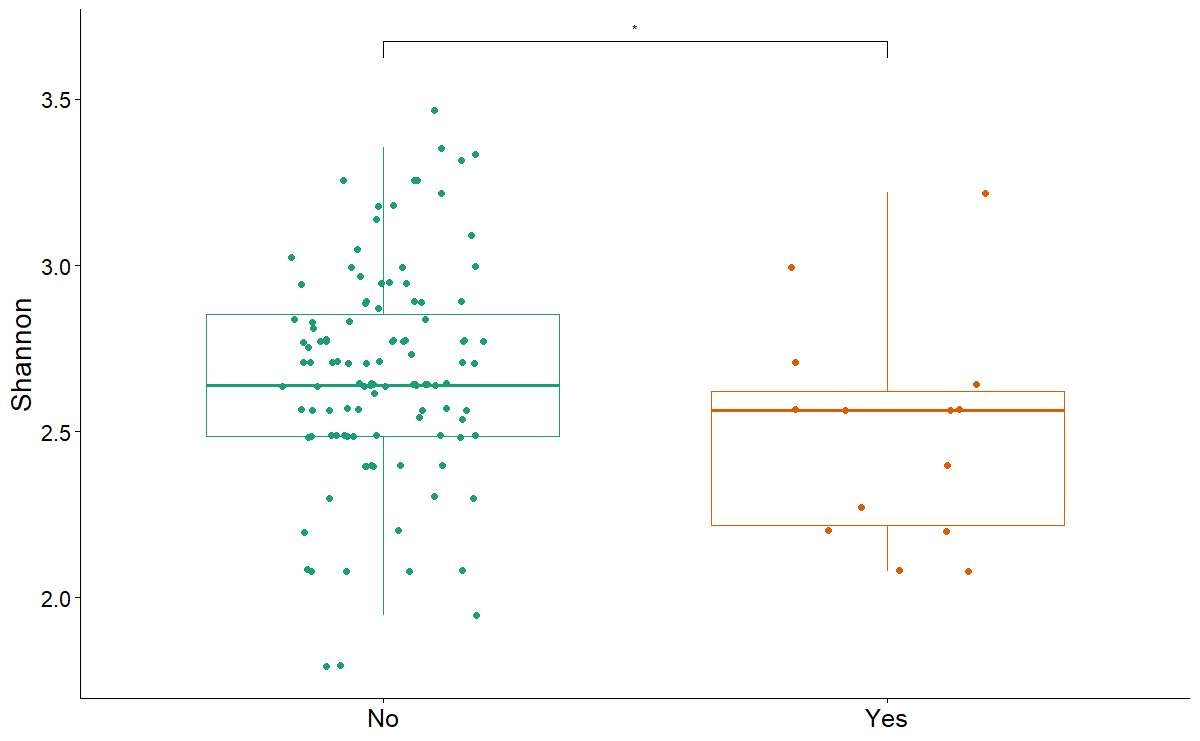


**Supplemental Figure 14.** Alpha diversity (Shannon index) of dogs reported to have recurrent gastrointestinal (GI) issues (“Yes”) and those without recurrent GI illness (“No”). * Pp<0.05, ** Pp<0.01, *** Pp<0.001

Differential abundance was determined by Random Forest to identify ARG abundance differences across and among groups. Many ARGs were also found to be differentially abundant by country of origin. The relative abundance of the ARGs *Inu(P)*, *tet(32)*, *aac(6’)-lm*, *aah(2”)-lla* was greater in dogs from the USA compared to all other countries of origin. The ARGs *tet(O)*, *mef(En2)*, *Inu(AN2)*, *tet(40)*, *Inu(C)*, and *cfxA6* were all more abundant in dogs from Israel compared to all other countries of origin (Supplemental Figure 15). The presence or absence of these ARGs could be the drivers of the difference in beta diversity that was observed among these populations.


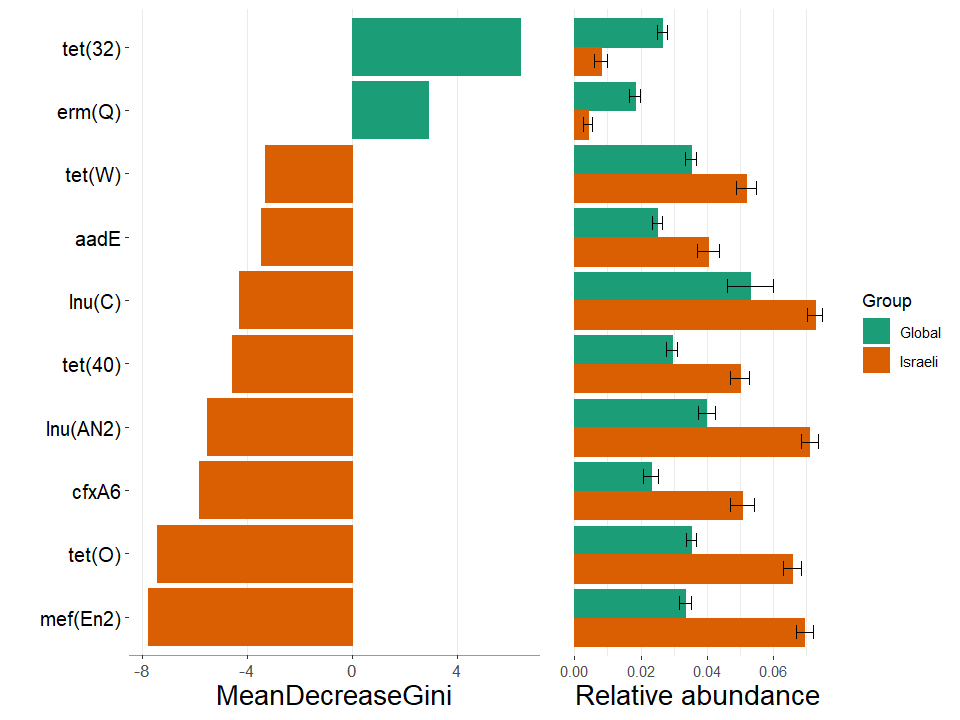


**Supplemental Figure 15.** Random forest analysis identifies a number of ARGs that are differentially abundant across global and Israeli dog populations.

*Differential Abundance of Israeli canine resistomes*

Differential abundance was determined by Random Forest to identify ARG abundance differences across demographic groups within the Israeli canines (Supplemental Figure 16). In dogs with recurrent gastrointestinal issues, several ARGs, including *aadS* and *mef(En2)*, and several lincosamide and tetracycline genes, had greater relative abundance, whereas *tetA(P)* was more abundant in the Israeli dog population without recurrent GI issues. Many ARGs were also differentially abundant by job type. In dogs that are sterilized, all differentially abundant ARGs had greater relative abundance compared to dogs that were not sterilized with the exception of *cfxA6*.

A
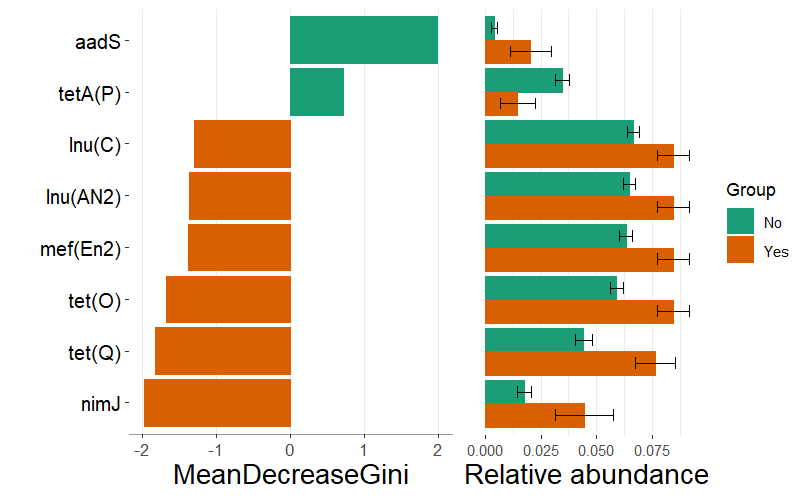


B
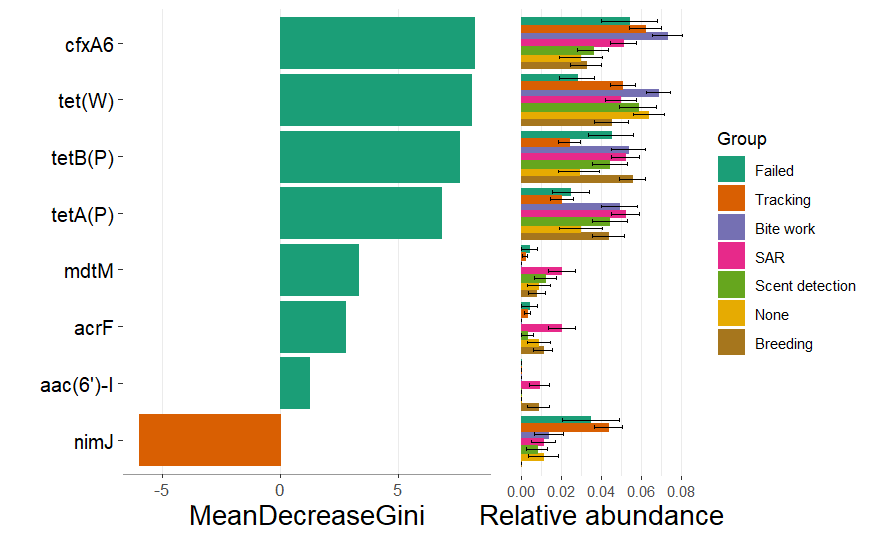


C
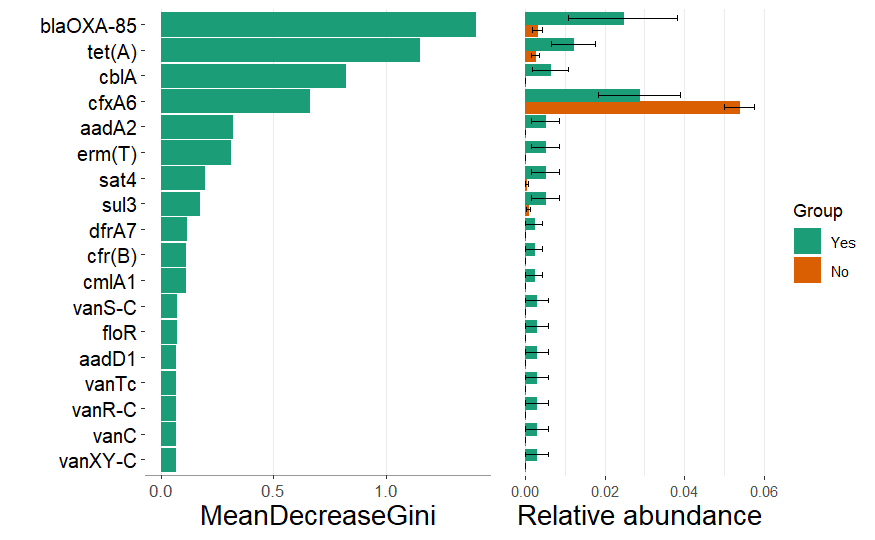


**Supplemental Figure 16.** Random forest analysis identifies a number of ARGs that are differentially abundant across Israeli dogs based on a) recurrent gastrointestinal issues, b) job type, and c) sterilization status.

*Plasmid analysis*

**Supplemental Table 7**: Frequency and percent of detection of various *lnc* plasmid types in domestic canine metagenomes

| lnu Group | Frequency | % of lnc family detects |
| --- | --- | --- |
| IncF | 308 | 60.0 |
| IncQ | 60 | 11.7 |
| IncX | 45 | 8.8 |
| IncI | 36 | 7.0 |
| IncH | 17 | 3.3 |
| IncY | 14 | 2.7 |
| IncN | 11 | 2.1 |
| IncB/O/K/Z | 7 | 1.4 |
| IncR | 7 | 1.4 |
| IncP | 5 | 1.0 |
| IncU | 3 | 0.6 |

**Supplemental Table 9**: Metagenome assembled genomics (MAGs) associated with antimicrobial resistance genes (ARGs) by country of dog’s origin, compared to overall sample population


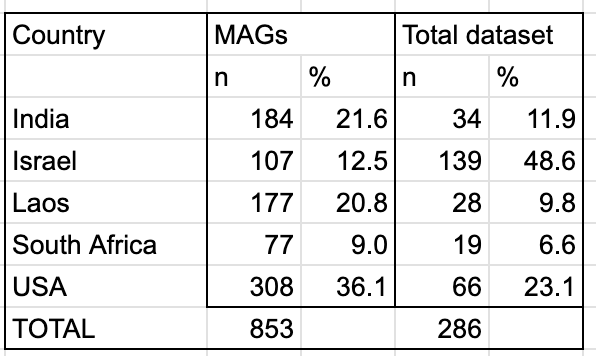


**Supplemental Figure 17**: Antimicrobial resistance genes (ARGs) identified on metagenome assembled genomics (MAGs) by antimicrobial subclass


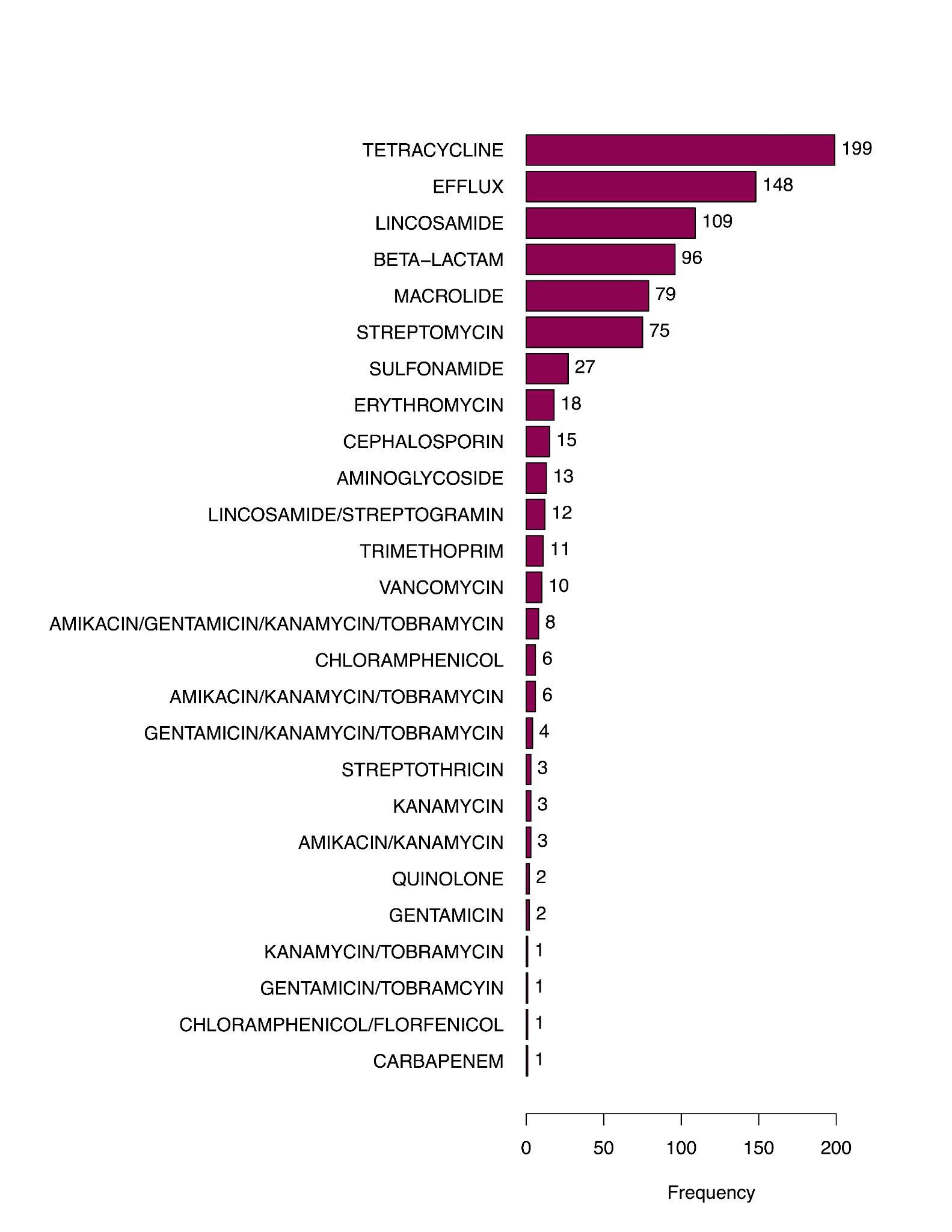


**Supplemental Figure 18**: Heatmap of genes within antimicrobial resistance classes by genus of the metagenome assembled genome, within a global canine dataset


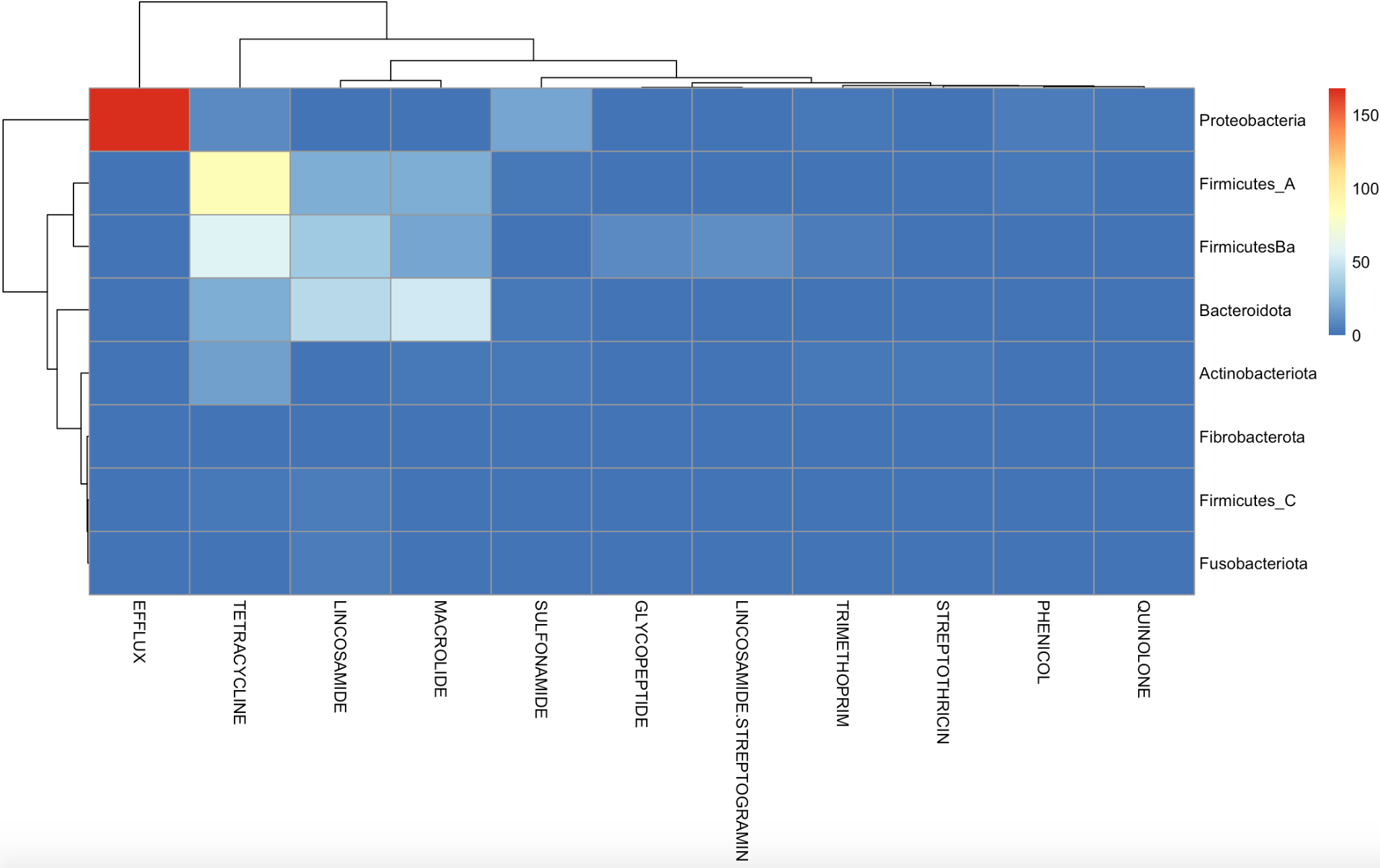


*Supplemental Citations*

1. Lapid, Roi, Yair Motro, Hillary Craddock, Boris Khalfin, Roni King, Gila Kahila Bar-Gal, and Jacob Moran-Gilad. 2023. “Fecal Microbiota of the Synanthropic Golden Jackal (Canis Aureus).” *Animal Microbiome* 5 (1): 37.
2. Wood, Derrick E., Jennifer Lu, and Ben Langmead. 2019. “Improved Metagenomic Analysis with Kraken 2.” *Genome Biology* 20 (1): 257.
3. Wright, Robyn J., Andrè M. Comeau, and Morgan G. I. Langille. 2023. “From Defaults to Databases: Parameter and Database Choice Dramatically Impact the Performance of Metagenomic Taxonomic Classification Tools.” *Microbial Genomics* 9 (3). https://doi.org/10.1099/mgen.0.000949.
4. Shen, Wei, and Hong Ren. 2021. “TaxonKit: A Practical and Efficient NCBI Taxonomy Toolkit.” *Journal of Genetics and Genomics = Yi Chuan Xue Bao* 48 (9): 844–50.
5. Lu, Jennifer, Natalia Rincon, Derrick E. Wood, Florian P. Breitwieser, Christopher Pockrandt, Ben Langmead, Steven L. Salzberg, and Martin Steinegger. 2022. “Metagenome Analysis Using the Kraken Software Suite.” *Nature Protocols* 17 (12): 2815–39.
6. Yin, Xiaole, Xiawan Zheng, Liguan Li, An-Ni Zhang, Xiao-Tao Jiang, and Tong Zhang. 2022. “ARGs-OAP v3.0: Antibiotic-Resistance Gene Database Curation and Analysis Pipeline Optimization.” *Proceedings of the Estonian Academy of Sciences: Engineering*, December. https://doi.org/10.1016/j.eng.2022.10.011.
7. Li, Dinghua, Chi-Man Liu, Ruibang Luo, Kunihiko Sadakane, and Tak-Wah Lam. 2015. “MEGAHIT: An Ultra-Fast Single-Node Solution for Large and Complex Metagenomics Assembly via Succinct de Bruijn Graph.” *Bioinformatics*  31 (10): 1674–76.
8. Feldgarden, Michael, Vyacheslav Brover, Narjol Gonzalez-Escalona, Jonathan G. Frye, Julie Haendiges, Daniel H. Haft, Maria Hoffmann, et al. 2021. “AMRFinderPlus and the Reference Gene Catalog Facilitate Examination of the Genomic Links among Antimicrobial Resistance, Stress Response, and Virulence.” *Scientific Reports* 11 (1): 12728.
9. Alcock, Brian P., William Huynh, Romeo Chalil, Keaton W. Smith, Amogelang R. Raphenya, Mateusz A. Wlodarski, Arman Edalatmand, et al. 2023. “CARD 2023: Expanded Curation, Support for Machine Learning, and Resistome Prediction at the Comprehensive Antibiotic Resistance Database.” *Nucleic Acids Research* 51 (D1): D690–99.
10. Doster, Enrique, Steven M. Lakin, Christopher J. Dean, Cory Wolfe, Jared G. Young, Christina Boucher, Keith E. Belk, Noelle R. Noyes, and Paul S. Morley. 2019. “MEGARes 2.0: A Database for Classification of Antimicrobial Drug, Biocide and Metal Resistance Determinants in Metagenomic Sequence Data.” *Nucleic Acids Research* 48 (D1): D561–69.
11. Carattoli, Alessandra, Ea Zankari, Aurora García-Fernández, Mette Voldby Larsen, Ole Lund, Laura Villa, Frank Møller Aarestrup, and Henrik Hasman. 2014. “In Silico Detection and Typing of Plasmids Using PlasmidFinder and Plasmid Multilocus Sequence Typing.” *Antimicrobial Agents and Chemotherapy* 58 (7): 3895–3903.
12. Pan, Shaojun, Chengkai Zhu, Xing-Ming Zhao, and Luis Pedro Coelho. 2022. “A Deep Siamese Neural Network Improves Metagenome-Assembled Genomes in Microbiome Datasets across Different Environments.” *Nature Communications* 13 (1): 2326.
13. Li, Heng. 2018. “Minimap2: Pairwise Alignment for Nucleotide Sequences.” *Bioinformatics*  34 (18): 3094–3100.
14. Danecek, Petr, James K. Bonfield, Jennifer Liddle, John Marshall, Valeriu Ohan, Martin O. Pollard, Andrew Whitwham, et al. 2021. “Twelve Years of SAMtools and BCFtools.” *GigaScience* 10 (2). https://doi.org/10.1093/gigascience/giab008.
15. Chaumeil, Pierre-Alain, Aaron J. Mussig, Philip Hugenholtz, and Donovan H. Parks. 2022. “GTDB-Tk v2: Memory Friendly Classification with the Genome Taxonomy Database.” *Bioinformatics*  38 (23): 5315–16.
16. Parks, Donovan H., Michael Imelfort, Connor T. Skennerton, Philip Hugenholtz, and Gene W. Tyson. 2015. “CheckM: Assessing the Quality of Microbial Genomes Recovered from Isolates, Single Cells, and Metagenomes.” *Genome Research* 25 (7): 1043–55.
17. Orakov, Askarbek, Anthony Fullam, Luis Pedro Coelho, Supriya Khedkar, Damian Szklarczyk, Daniel R. Mende, Thomas S. B. Schmidt, and Peer Bork. 2021. “GUNC: Detection of Chimerism and Contamination in Prokaryotic Genomes.” *Genome Biology* 22 (1): 178.
18. Chan, Patricia P., Brian Y. Lin, Allysia J. Mak, and Todd M. Lowe. 2021. “TRNAscan-SE 2.0: Improved Detection and Functional Classification of Transfer RNA Genes.” *Nucleic Acids Research* 49 (16): 9077–96.
19. Albanese, Davide, and Claudio Donati. 2021. “Large-Scale Quality Assessment of Prokaryotic Genomes with Metashot/Prok-Quality.” *F1000Research* 10 (August): 822.
20. Olm, Matthew R., Christopher T. Brown, Brandon Brooks, and Jillian F. Banfield. 2017. “DRep: A Tool for Fast and Accurate Genomic Comparisons That Enables Improved Genome Recovery from Metagenomes through de-Replication.” *The ISME Journal* 11 (12): 2864–68.
21. Liu, Chi, Yaoming Cui, Xiangzhen Li, and Minjie Yao. 2021. “Microeco: An R Package for Data Mining in Microbial Community Ecology.” *FEMS Microbiology Ecology* 97 (2). https://doi.org/10.1093/femsec/fiaa255.
22. Shannon, C. E. 1948. “A Mathematical Theory of Communication.” *The Bell System Technical Journal* 27 (3): 379–423.
23. Bray, J. Roger, and J. T. Curtis. 1957. “An Ordination of the Upland Forest Communities of Southern Wisconsin.” *Ecological Monographs* 27 (4): 325–49.
